# TBX2 controls a proproliferative gene expression program in melanoma

**DOI:** 10.1101/gad.348746.121

**Published:** 2021-12-01

**Authors:** Sizhu Lu, Pakavarin Louphrasitthiphol, Nishit Goradia, Jean-Philippe Lambert, Johannes Schmidt, Jagat Chauhan, Milap G. Rughani, Lionel Larue, Matthias Wilmanns, Colin R. Goding

**Affiliations:** 1 Ludwig Institute for Cancer Research, Nuffield Department of Clinical Medicine, University of Oxford, Headington, Oxford OX3 7DQ, United Kingdom;; 2 Department of Surgery, Faculty of Medicine, University of Tsukuba, Tsukuba, Ibaraki 305-8575, Japan;; 3 European Molecular Biology Laboratory, Hamburg Unit, 22607 Hamburg, Germany;; 4 Lunenfeld-Tanenbaum Research Institute, Mount Sinai Hospital, Toronto, Ontario M5G 1X5, Canada;; 5 Department of Molecular Medicine and Cancer Research Centre, Université Laval, Québec City, Québec G1R 3S3, Canada; CHU de Québec Research Center, Centre Hospitalier de l'Université Laval, Québec City, Québec G1V 4G2, Canada;; 6 Institut Curie, PSL Research University, U1021, Institut National de la Santé et de la Recherche Médicale, Normal and Pathological Development of Melanocytes, 91405 Orsay Cedex, France;; 7 Université Paris-Sud, Université Paris-Saclay, UMR 3347 Centre National de la Recherche Scientifique, 91405 Orsay Cedex, France;; 8 Equipe Labellisée Ligue Contre le Cancer, 91405 Orsay Cedex, France;; 9 University Hamburg Clinical Center Hamburg-Eppendorf, 20251 Hamburg, Germany

**Keywords:** TBX2, proliferation, senescence, cell cycle, DNA binding, DNA binding specificity

## Abstract

Senescence shapes embryonic development, plays a key role in aging, and is a critical barrier to cancer initiation, yet how senescence is regulated remains incompletely understood. TBX2 is an antisenescence T-box family transcription repressor implicated in embryonic development and cancer. However, the repertoire of TBX2 target genes, its cooperating partners, and how TBX2 promotes proliferation and senescence bypass are poorly understood. Here, using melanoma as a model, we show that TBX2 lies downstream from PI3K signaling and that TBX2 binds and is required for expression of E2F1, a key antisenescence cell cycle regulator. Remarkably, TBX2 binding in vivo is associated with CACGTG E-boxes, present in genes down-regulated by TBX2 depletion, more frequently than the consensus T-element DNA binding motif that is restricted to Tbx2 repressed genes. TBX2 is revealed to interact with a wide range of transcription factors and cofactors, including key components of the BCOR/PRC1.1 complex that are recruited by TBX2 to the *E2F1* locus. Our results provide key insights into how PI3K signaling modulates TBX2 function in cancer to drive proliferation.

## Abstract (precis)

In this study, Lu et al. investigated the repertoire of TBX2 (the antisenescence T-box family transcription repressor) target genes, its cooperating partners, and how TBX2 promotes proliferation and senescence bypass. Using melanoma as a model, they show that TBX2 lies downstream from PI3K signaling and that TBX2 binds and is required for expression of E2F1, a key antisenescence cell cycle regulator.

Senescence is characterized as an irreversible replicative arrest associated with activation of cell cycle inhibitors, chromatin reorganization, and inflammatory signaling known as the senescence-associated secretory phenotype, and can be triggered by a wide variety of cell-extrinsic and -intrinsic signals (for reviews, see Kuilman et al. 2010; Campisi 2013; He and Sharpless 2017; Herranz and Gil 2018). Once considered an artifact of culture conditions, senescence is now increasingly recognized as a physiologically important biological process. For example, senescence is critical for correct embryonic development (Muñoz-Espín et al. 2013; Storer et al. 2013), but as organisms age, the numbers of senescent cells can increase, leading to local inflammation, fibrosis, and stem cell dysfunction, which contribute to a wide range of age-related diseases (Campisi and Robert 2014; McHugh and Gil 2018; Khosla et al. 2020). This has led to significant interest in the possibility of targeting senescence using so-called senolytic therapy as an antiaging strategy (Zhu et al. 2015; Pignolo et al. 2020).

The negative role of senescence in aging is counterbalanced by its critical role in protecting against cancer initiation (Braig et al. 2005; Chen et al. 2005; Michaloglou et al. 2005). Activation of oncogenes such as RAS or BRAF can drive cells to undergo replicative stress (Bartkova et al. 2006; Di Micco et al. 2006), leading to oncogene-induced senescence (OIS) (Serrano et al. 1997). Consequently, for cancer initiation to occur, activation of oncogenes must be accompanied by senescence bypass mediated by inactivation of tumor suppressors such as Rb1—directly or via inactivation of INK4a (*CDKN2a*)—and p53.

One of the best-characterized models for understanding the role of senescence in cancer is cutaneous melanoma (Bennett 2015). For example, activation of BRAF in melanocytes can trigger an initial proliferative event followed by p16^INK4a^-triggered senescence, leading to formation of a benign nevus (Michaloglou et al. 2005; Gray-Schopfer et al. 2006). Progression to a radial growth phase melanoma requires inactivation of the Rb1 pathway that can occur via several mechanisms that converge on factors upstream of or downstream from Rb1 or p53 (Bennett 2015). This is reflected in mouse models, where melanocyte-specific activation of BRAF or NRAS is insufficient to generate melanomas but can do so when combined with loss of *INK4a* by mutation (Ackermann et al. 2005; Dhomen et al. 2009; Goel et al. 2009; Burd et al. 2014; Damsky et al. 2015) or its silencing by activated β-catenin (Delmas et al. 2007). Similarly, in zebrafish, activation of BRAF induces nevi, but will generate melanoma when combined with inactivation of p53 (Patton et al. 2005).

In addition to inactivation of the p53 and Rb1 pathways, OIS in melanoma and other cancers can be bypassed via activation of PI3K signaling, most frequently via inactivation of *PTEN* (Dankort et al. 2009; Vredeveld et al. 2012; Conde-Perez et al. 2015). However, how activation of PI3K signaling drives senescence bypass is not well understood. In primary cells, activation of PI3K can promote senescence, in part by promoting mTORC1-driven protein synthesis, leading to ER stress (Alimonti et al. 2010). In contrast, in melanoma cells with activated BRAF, it is possible that constitutive activation of PI3K may facilitate senescence bypass through the same mechanism, increasing capacity for protein synthesis to meet the demands of proliferation driven by constitutive MAPK signaling. Alternatively, PI3K signaling could increase the activity of transcription factors that facilitate senescence bypass. One candidate is TBX2, a key developmental transcription repressor (Naiche et al. 2005; Abrahams et al. 2010; Ghosh et al. 2017) that was identified in a senescence bypass screen in breast cancer, where it was shown to suppress p16^INK4A^ (*CDKN2A*) expression (Jacobs et al. 2000). In contrast, in melanoma, where p16^INK4A^ is frequently inactivated by mutation, depletion of TBX2 in both mouse and human melanoma cells leads to senescence associated with increased expression of *CDKN1A* encoding the p21 cyclin-dependent kinase inhibitor (CDKi), with TBX2 able to directly bind and repress the *CDKN1A* promoter (Prince et al. 2004; Vance et al. 2005). Although in rhabdomyosarcoma TBX2 can repress *PTEN* (Zhu et al. 2016), a negative PI3K regulator, whether it is activated by PI3K signaling is unknown. However, while TBX2 can suppress cell cycle regulators associated with senescence, whether it plays a wider role in reprogramming gene expression is unclear, as few target genes have been robustly defined.

Like other T-box factors, TBX2 will make DNA contact in both the major and minor grooves (Müller and Herrmann 1997; Coll et al. 2002; El Omari et al. 2012). However, while minor groove interaction with two G residues is critical for sequence recognition, they are insufficient to provide target gene specificity. Although TBX2 can recognize nucleosomal DNA (Demay et al. 2007; Zhu et al. 2018), suggesting it may play a role as a pioneer transcription factor (Zaret and Mango 2016), sequence-specific binding presumably arises via cofactor interaction. However, the repertoire of TBX2-interacting factors is poorly delineated, and whether in vivo it binds sequences other than the consensus T-element AGGTGTGA is unclear. Here we address these key outstanding questions to reveal that TBX2 is up-regulated by PI3K signaling, and find surprisingly that TBX2 depletion leads to loss of expression of many TBX2-bound genes, including *E2F1*. Remarkably, TBX2 is associated in vivo with E-boxes as well as T-elements and interacts with a wide range of transcription factors and cofactors, including the BCOR/PRC11 complex.

## Results

### TBX2 is regulated by PI3K signaling

**Figure 1.**
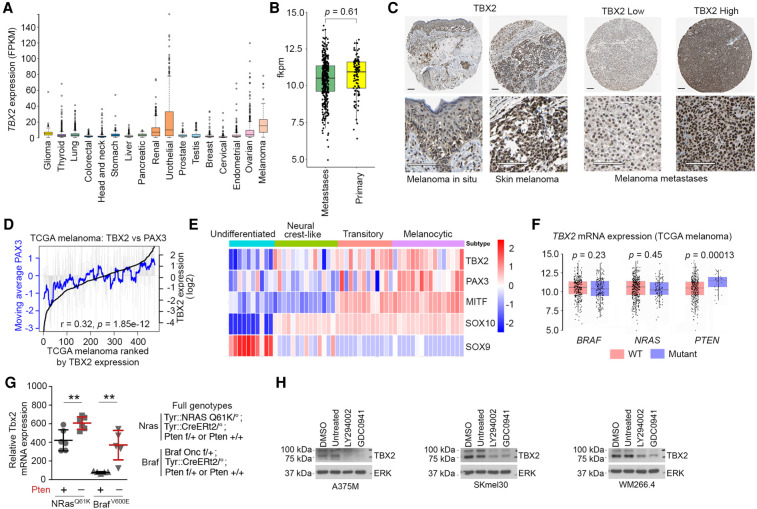
Regulation of *TBX2* by PI3K signaling. (*A*) TBX2 mRNA expression from the TCGA in range of cancers. (*B*) Relative expression of Tbx2 mRNA in primary or metastatic TCGA melanomas. (*C*) Immunohistochemistry showing TBX2 expression in examples of human melanomas. Scale bars, 100 mm. (*D*) Expression of *PAX3* and *TBX2* in TCGA melanoma cohort ranked by *TBX2* expression (black line). Gray bars indicate *PAX3* expression in each individual tumor, and the gray line indicates moving average over each group of 20 samples. (*E*) Relative expression of the indicated genes in 53 cell lines grouped by phenotype. Data are from Tsoi et al. (2018). (*F*) TBX2 mRNA expression in TCGA melanoma comparing WT with mutated BRAF, NRAS, or PTEN. (*G*) Expression of *Tbx2* mRNA in mouse melanoma tumors within the indicated genotypes. *NRas*^Q61K^/*Pten*^+^, *n* − 8; *NRas*^Q61K^/*Pten*^–^, *n* = 6; *BRaf*^V600E^/*Pten*^+^, *n* = 5; *BRaf*^V600E^/*Pten*^–^, *n* = 5. Error bars indicate SEM; Student's *t*-test. (*H*) Western blot showing expression of TBX2 in the indicated human melanoma cells after treatment with 20 mM LY294002 or 5 mM GDC0941 for 24 h. ERK was used as a loading control.

TBX2 is a key antisenescence transcription factor whose role and regulation are poorly understood. After urothelial cancer, melanoma exhibits the second highest levels of TBX2 mRNA among all cancers (Fig. 1A). However, examination of *TBX2* expression in melanoma metastases versus primary tumors indicates no significant difference, but in each group there is a wide range of *TBX2* mRNA expression (Fig. 1B), most likely reflecting the importance of the intratumor microenvironment in controlling *TBX2* activity. This conclusion was strengthened by examining individual melanoma tumors that indicated that TBX2 protein expression is highly variable between tumors (Fig. 1C) and that *TBX2* mRNA levels vary significantly between individual tumors in the TCGA melanoma cohort (Fig. 1D, black line). In part, this may be because *TBX2* can be regulated by PAX3 (Liu et al. 2013), a key melanocyte transcription factor that can promote a melanogenic gene expression program but that also prevents differentiation (Lang et al. 2005), and potentially by the microphthalmia-associated transcription factor MITF (Carreira et al. 2000; Béjar et al. 2003), a key regulator of melanoma biology (Goding and Arnheiter 2019). In agreement, *TBX2* expression followed closely that of *PAX3* in human melanomas (Fig. 1D). Since in vivo tumors contain a mix of melanoma and nonmelanoma cells, we also asked whether any correlation with PAX3 or MITF was observed in 53 melanoma cell lines grouped according to four different melanoma phenotypes distinguished by *SOX10*, *SOX9*, and *MITF* expression (Tsoi et al. 2018). The results (Fig. 1E) show that *TBX2* is predominantly expressed in MITF^High^ cell lines corresponding to the differentiated and transitory phenotypes, and especially in the PAX3^High^ melanocytic phenotype.

*PAX3* expression is controlled in melanoma by PI3K signaling (Bonvin et al. 2012), which has been implicated in both melanomagenesis and senescence bypass through inactivation or loss of PTEN, an inhibitor of PI3K, or via activating mutations in PI3K itself (Dankort et al. 2009; Madhunapantula and Robertson 2009; Cao et al. 2013; Marsh Durban et al. 2013). These observations raised the possibility that *TBX2* expression might be controlled by PI3K activity. In human melanomas, no correlation was observed between *TBX2* expression and activating mutation of BRAF or NRAS, but, in contrast, inactivation of *PTEN* correlated with elevated *TBX2* expression compared with tumors expressing WT *PTEN* (Fig. 1F). Beyond the key driver mutations, human melanomas are genetically complex. To be sure that loss of PTEN led to elevated TBX2 expression, we also examined *Tbx2* mRNA levels in tumors from genetically defined mouse models. The results revealed that mouse melanomas with activated Nras, which can activate PI3K signaling, expressed higher levels of Tbx2 than those with the *Braf*^*V600E*^ mutation, and that in both *Nras*^*Q61K*^ or *Braf*^*V600E*^ melanomas, loss of *Pten* increased *Tbx2* expression (Fig. 1G). Consistent with the correlations between mutations activating PI3K signaling and *TBX2* in human and mouse tumors, TBX2 protein levels were decreased in three human melanoma cell lines using two different PI3K inhibitors: LY294002 or GDC0941 (Fig. 1H). Similar results were obtained on inhibition of AKT that lies downstream from PI3K signaling using p-GSK3 as a marker of PI3K pathway activity (Supplemental Fig. S1A). The change in TBX2 protein was mirrored by a loss of TBX2 mRNA expression following PI3K inhibition (Supplemental Fig. S1B). Collectively, these observations place the antisenescence transcription factor *TBX2* downstream from PI3K signaling that mediates senescence bypass in BRAF mutated melanoma.

### HA epitope tagging endogenous Tbx2

**Figure 2.**
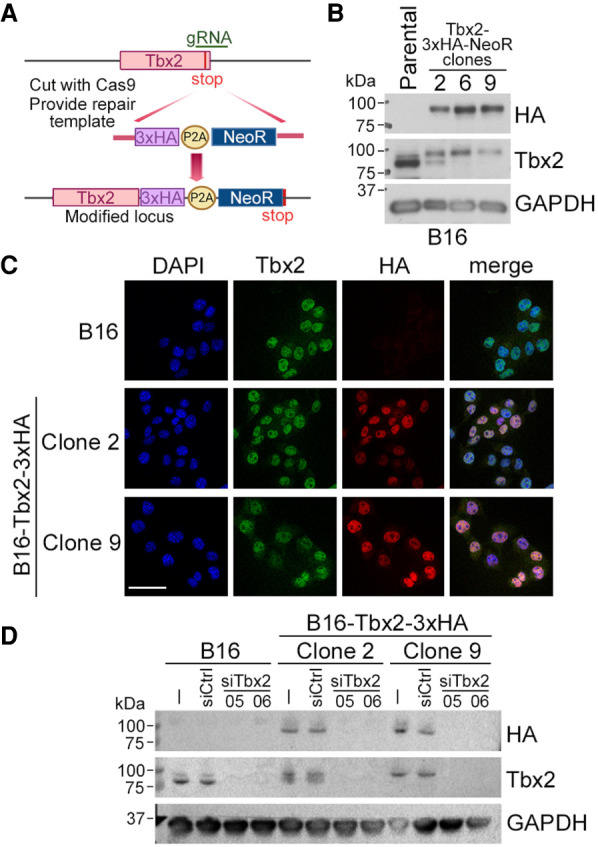
C-terminal epitope tagging endogenous Tbx2. (*A*) Strategy to tag endogenous Tbx2 with a 3x HA epitope tag. P2A indicates a self-cleaving peptide. (*B*) Western blot showing parental B16 cells and three selected Tbx2-HA-tagged derivative cell lines. Gapdh was used as a loading control. (*C*) Immunofluorescence of parental B16 cells and two selected Tbx2-H-expressing cell lines using anti-Tbx2 and anti-HA antibodies as indicated. Scale bar, 50 μm. (*D*) Western blot of parental B16 cells and Tbx2-HA-expressing derivatives transfected with two different Tbx2 siRNAs.

To gain an insight into the role of TBX2 in melanoma, we aimed to identify the repertoire of TBX2-bound target genes using chromatin immunoprecipitation followed by high-throughput sequencing (ChIP-seq). To this end, we initially attempted to undertake a ChIP-seq experiment using commercially available antibodies, but found that they were unable to immunoprecipitate chromatin-bound TBX2 to high efficiency. This is consistent with a previous study (Decaesteker et al. 2018) in which Tbx2 ChIP-seq from neuroblastoma cells identified only 557 significant bound sites (*q* < 0.05). Of these, only 107 could be converted to mouse genomic locations, none of which overlapped with those identified in a more recent ChIP-seq data set from developing mouse lungs (Lüdtke et al. 2021). These studies highlight the need for a robust and reproducible approach to Tbx2 ChIP. As an alternative strategy to using antibody against endogenous protein that can suffer from low affinity or lack of specificity, we previously used HA epitope-tagged proteins expressed from an inducible vector to generate high-quality ChIP-seq data sets (see Louphrasitthiphol et al. 2020). However, since increasing expression of a transcription factor can lead to more peaks being called (Louphrasitthiphol et al. 2020), we chose instead to tag the endogenous gene with the HA epitope using the endogenous fluorescent tagging (EFLUT) CRISPR/Cas9 system as described (Stewart-Ornstein and Lahav 2016). To this end, we designed guide RNAs to facilitate insertion of sequences encoding a 3x HA epitope tag, a P2A self-cleaving peptide, and a neomycin resistance immediately before the *Tbx2* stop codon (Fig. 2A). For this, we chose the B16 mouse melanoma cell line because it is readily susceptible to senescence after Tbx2 depletion (Prince et al. 2004; Vance et al. 2005) and does not express Tbx3 (Vance et al. 2005), a highly related T-box factor expressed in many human melanoma cell lines (Rodriguez et al. 2008) that can potentially regulate similar genes to Tbx2 (Hoogaars et al. 2008). After transfection of the appropriate template and guide RNAs, clones were selected using neomycin and screened by genomic PCR (Supplemental Fig. S2A,B). Three clones were identified (clones 2, 6, and 9), with Western blotting (Fig. 2B) indicating that all expressed HA-tagged Tbx2, although clone 2 appeared to retain some untagged Tbx2. Clones 2 and 9 exhibited a PCR profile consistent with the HA-P2A-Neo cassette being inserted correctly into at least one copy of the Tbx2 locus (Supplemental Fig. S2B), whereas clone 6 appeared to have an aberrant PCR profile using primers F2/R3. To verify the insertion was correct, PCR products from clones 2 and 9 were sequenced, and the results confirmed that the Tbx2 stop codon was deleted and the HA tag was in-frame with the C terminus of Tbx2 (Supplemental Fig. S2C). Immunofluorescence confirmed that, like endogenous Tbx2, the HA-tagged protein was localized to the nucleus (Fig. 2C), and two different siRNAs specific for Tbx2 were able to deplete both the endogenous protein and the HA-tagged versions (Fig. 2D).

### Tbx2 interacts with non-T-element targets

**Figure 3.**
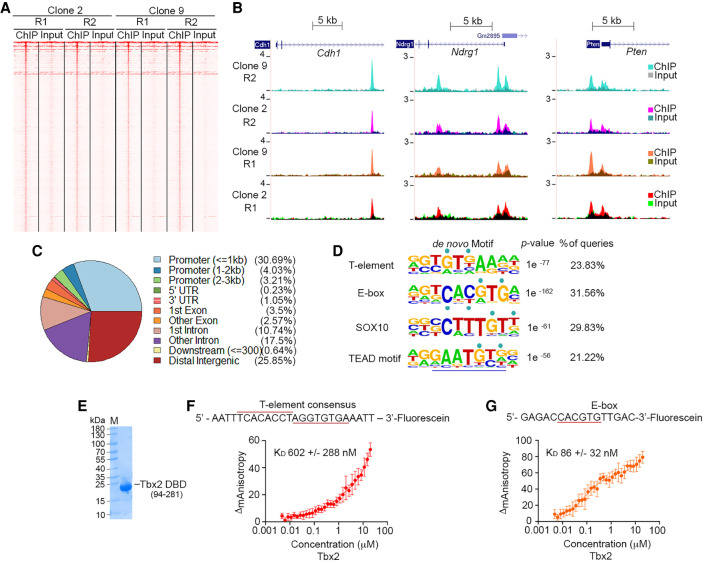
Tbx2-bound genes. (*A*) Read density heat maps derived from biological replicate (R1 and R2) ChIP-seq assays using anti-HA antibody and Tbx2-HA clones 2 and 9 compared with corresponding input controls. (*B*) UCSC browser screenshots showing ChIP peaks at the *Cdh1*, *Ndrg1*, and *Pten* genes. Reads corresponding to immunoprecipitated DNA are overlaid on those from the corresponding input controls. (*C*) Pie chart showing the relative genomic distribution of the Tbx2-HA ChIP peaks. (*D*) Consensus binding motifs beneath the Tbx2-HA ChIP peaks. (*E*) Coomassie blue staining of bacterially expressed and purified Tbx2 T-box (amino acids 94–281) (*F*,*G*) Fluorescence anisotropy using increasing concentrations of purified Tbx2 T-box and the indicated fluorescein labeled T-element (*F*) or E-box (*G*). Error bars indicate SD; *n* = 3.

We next identified the repertoire of genes bound by ChIP-seq of HA-tagged endogenous Tbx2. To ensure reproducibility, we performed biological replicates on both clone 2 and clone 9 (Supplemental Fig. S3A) and compared with the input controls. A read density heat map of the results (Fig. 3A) shows similar binding profiles using a peak-calling *q*-value of 0.01. For example, 2623 of the peaks called were common between the replicates of clone 2, 2682 were common between replicates of clone 9, and 1705 were common between all four replicates (Supplemental Fig. S3B). Of note, few peaks overlapped between our data sets and that previously published for mouse lungs (Supplemental Fig. S3C; Lüdtke et al. 2021), possibly because the genomic distribution of Tbx2 in lungs and melanoma are different, or because immunoprecipitation with anti-Tbx2 antibody may be less specific compared with use of anti-HA antibody. Moreover, because the previous study did not generate replicate ChIPs, we were uncertain of the reproducibility of the Tbx2 binding sites identified. Given that we used two clones each in replicate, we are confident that the binding sites identified are robust, a conclusion confirmed since we detected binding to previously reported Tbx2 target genes *Cdh1* (Rodriguez et al. 2008), *Ndrg1* (Crawford et al. 2019), and *Pten* (Fig. 3B; Zhu et al. 2016). Notably, while the promoter of *Cdh1* was previously reported to be a Tbx2 target (Rodriguez et al. 2008), the ChIP-seq results indicate that binding was within intron 2. Nevertheless binding to the *Pten* promoter, previously reported in muscle (Zhu et al. 2016), was confirmed, and the reported binding of Tbx2 to the proximal promoter of *Ndrg1* in breast cancer cells (Crawford et al. 2019) was also found, although with additional sites occupied within the first intron. A list of Tbx2-bound genes is shown in Supplemental Table S1. Overall, 38% of binding sites identified were within 3 kb upstream of the transcription start site, and of these, the great majority were located in the proximal promoter (Fig. 3C). Some 26% of Tbx2 binding sites were located in intergenic regions, and a similar proportion was found in introns. Gene ontology analysis of the binding sites ranked by *P*-value is shown in Supplemental Figure S3D and reveals that intronic or intergenic binding is connected to developmental processes. In contrast, those genes where binding is associated with promoters are preferentially linked to metabolism.

The consensus binding site for T-box factors has been identified using in vitro binding assays and binding site selection experiments to be AGGTGTGA (Kispert and Herrmann 1993; Carreira et al. 1998; Conlon et al. 2001). DNA-T-box cocrystal structures (Müller and Herrmann 1997; Coll et al. 2002; El Omari et al. 2012) revealed binding to a widened minor groove and base-specific contacts with the Gs at positions 3 and 5 in the consensus. Consistent with this, analysis of the sequences beneath the Tbx2 peaks revealed a near consensus motif G/A**G**T**G**TGA (Fig. 3D). Surprisingly, however, we found Tbx2 could also recognize an E-box motif, TCACGTG, including a 5′ T residue with a lower *P*-value than the T-element. This sequence represents a consensus for recognition by the basic helix–loop–helix leucine zipper (bHLH-LZ) microphthalmia-associated transcription factor MITF (Aksan and Goding 1998), which controls many aspects of melanocyte development and melanoma biology (Goding and Arnheiter 2019), as well as by the bHLH-LZ transcription factors USF1 and USF2, which are implicated in control of expression of a wide range of E-box-containing genes (Corre and Galibert 2005). In addition to the E-box element, additional motifs were identified corresponding to those bound by the transcription factors SOX10 and TEAD, which, like the T-element and E-box, contained the key GTG recognition sequence. SOX10 is preferentially expressed in the neural crest, transitory and melanocytic phenotypes (Fig. 1E) and plays a key role in melanocyte and melanoma biology (Seberg et al. 2017a), while the TEAD transcription factor lies downstream from the Hippo signaling pathway implicated in response to mechanical stress and control of organ size and apoptosis (Ma et al. 2019).

Using a bacterially expressed and purified Tbx2 DNA-binding domain (Fig. 3E), we determined using fluorescent anisotropy that the in vitro DNA binding affinity of Tbx2 for the T-element (Fig. 3F) and the E-box (Fig. 3G) were 602 nM and 86 nM, respectively. The high affinity for the E-box compared with the T-element was surprising. We therefore examined binding to both the TEAD and SOX motifs as well as a GATA element that did not feature in the list of Tbx2 targets identified in our ChIP-seq data set. The results (Supplemental Fig. S3E) indicated that the affinity of Tbx2 binding to all three motifs was between that of T-element and the E-box, ranging from 292 nM to 173 nM. Collectively, these results may indicate that Tbx2 binding in vitro is relatively nonspecific, but that in vivo specificity is likely achieved through interaction with specific cofactors (see below).

### Tbx2-mediated gene regulation

**Figure 4.**
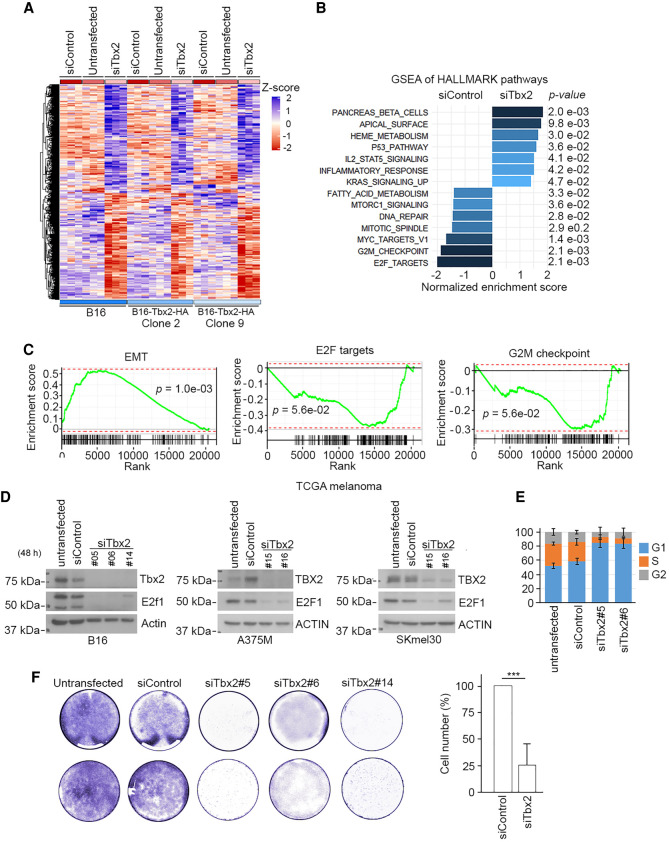
Tbx2-dependent gene expression. (*A*) Heat map showing changes in gene expression derived from biological triplicate RNA-seq analysis of untransfected parental B16 cells or the Tbx2-HA derivative clones 2 and 9 compared with cells transfected with control or Tbx2-specifc siRNA. (*B*) GSEA from B16 cells derived by comparing expression of cells transfected with siControl versus those transfected with Tbx2-specific siRNA. (*C*) GSEA derived from the TCGA melanoma cohort by comparing gene expression between the 50 tumors with the highest Tbx2 expression versus the 50 with the lowest expression. (*D*) Western blot of B16 and A375M cells after siRNA-mediated depletion of TBX2. (*E*) Cell cycle profiles determined using flow cytometry of cells 48 h after depletion of Tbx2. Error bars indicate SD. *N* = 3. (*F*) Cell proliferation assay using crystal violet staining using B16 cells performed 96 h after depletion of TBX2 using the indicated siRNAs.

While transcription factor binding to specific sequence elements is required for transcription regulation, it is not necessarily sufficient. A ChIP-seq peak, for example, cannot distinguish between many short-lived transcriptionally nonproductive binding events and fewer long-lived binding events reflecting increased dwell time that may lead to changes in transcriptional output (Lickwar et al. 2012). We therefore sought to determine the repertoire of genes regulated by Tbx2 using siRNA to deplete Tbx2 in parental B16 cells, as well as clones 2 and 9 expressing HA-tagged Tbx2 (Supplemental Fig. S4A) followed by RNA sequencing. The experiment was performed in biological triplicate, and a heat map corresponding to the significantly (*P* ≤ 0.05) differentially expressed genes (DEGs) on Tbx2 depletion in all three isogenic cell lines is shown in Figure 4A, with principle component analysis shown in Supplemental Figure S4B. A total of 4238 DEGs was identified following Tbx2 depletion in B16 cells, and 3507 and 3377 in clones 2 and 9, respectively. A full list of DEGs is provided in Supplemental Tables S2A and 2B. Depletion of Tbx2 in parental B16 cells led to >2149 genes being significantly up-regulated, consistent with Tbx2 playing a role as a transcriptional repressor, as reported (Carreira et al. 1998; Abrahams et al. 2010). However, 2089 genes were also down-regulated. Depletion of Tbx2 in parental B16 cells as well as in clones 2 and 9 revealed 1020 genes in common up-regulated after Tbx2 depletion and a further 1170 genes that were down-regulated (Supplemental Fig. S4C). Given the consensus that Tbx2 is a transcriptional repressor, the down-regulation of many genes observed could arise through indirect regulation. However, this observation also raised the possibility that in some circumstances Tbx2 may facilitate transcription activation.

Gene set enrichment analysis (GSEA) of DEGs from B16 cells depleted for Tbx2 revealed a decrease in expression of targets associated with mTORC1 signaling as well as the transcription factors Myc and E2f1 (Fig. 4B), consistent with Tbx2 promoting proliferation. An increase was also observed in the p53 pathway, indicative of cellular stress and inflammatory signaling. GSEA after depletion of Tbx2 in clones 2 and 9 (Supplemental Fig. S4D) revealed that the deregulated genes were associated with biological processes similar to those identified using parental B16 cells (Fig. 4B). A reduction in proliferation together with activation of the p53 pathway and inflammatory signaling is a hallmark of senescence and would be consistent with previous work showing that depletion of Tbx2 causes senescence in fibroblasts as well as in B16 and human melanoma cell lines (Jacobs et al. 2000; Prince et al. 2004; Vance et al. 2005). To determine whether TBX2 in human tumors was likely to perform a similar role, we performed GSEA on the TCGA melanoma cohort ranked according to TBX2 expression and asked which gene sets were enriched in the 50 highest TBX2-expressing tumors compared with the 50 lowest. The results (Fig. 4C) were broadly similar to those obtained using siRNA-mediated Tbx2 depletion in B16 cells, with low Tbx2-expressing tumors being enriched in EMT-expressing genes, while those with high Tbx2 expression were enriched in gene sets associated with E2F targets and the G2M checkpoint. Consistent with the GSEA, Tbx2 depletion using three different siRNAs in mouse B16 melanoma cells led to a strong reduction of E2f1 protein expression (Fig. 4D). These results were not restricted to mouse B16 cells, as similar observations were made in the A375M and SKmel30 human melanoma cell lines. These data strongly suggest that Tbx2 plays a key role in promoting cell cycle progression. To confirm this, we used independent siRNAs to deplete Tbx2 from B16 melanoma cells and used flow cytometry to compare their cell cycle profile with untransfected cells or those transfected with a control siRNA. The results (Fig. 4E) indicated a significant increase in the G1 population at the expense of S and G2, and a corresponding decrease in cell number (Fig. 4F).

### Tbx2 regulates genes coordinating the cell cycle

**Figure 5.**
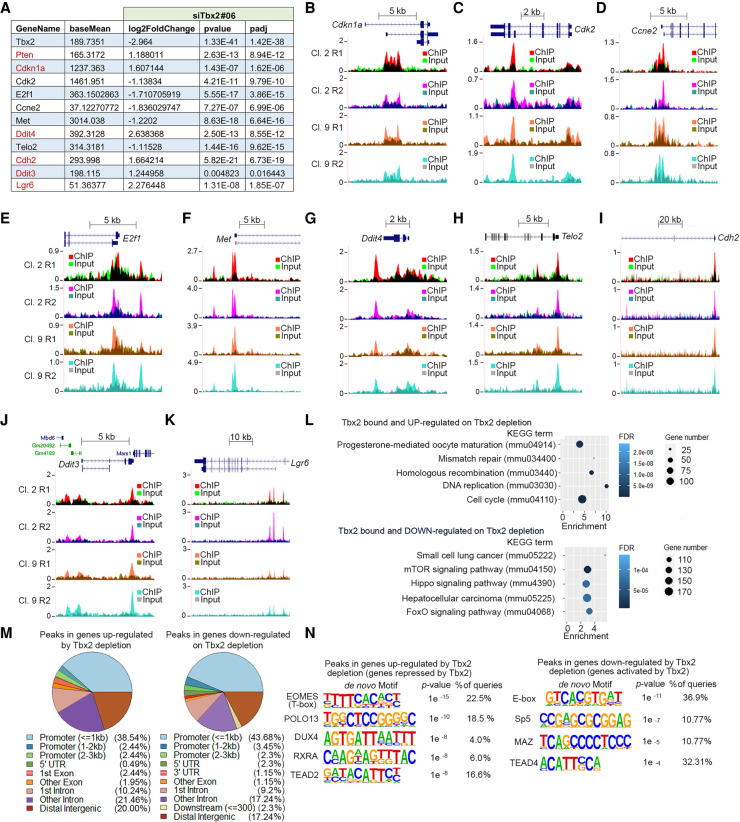
Identification of direct Tbx2 target genes. (*A*) Relative expression of the indicated genes that are directly bound by TBX2 in parental B16 cells or cells depleted for Tbx2 using siRNA. Expression of genes indicated in red increases on Tbx2 depletion and is therefore repressed by Tbx2, while those whose expression is decreased are activated by TBX2. (*B*–*K*) UCSC genome browser screenshots showing Tbx2 binding to the indicated loci derived from biological replicate ChIP-seq of Tbx2-HA-expressing clones 2 and 9 replicates R1 and R2. Reads obtained after HA immunoprecipitation are overlaid on the corresponding input controls. (*L*) KEGG analysis of Tbx2-bound and -regulated genes showing the top five terms for the up-regulated and down-regulated genes. (*M*) Pie charts showing genomic distribution of Tbx2 binding in Tbx2-repressed and Tbx2-activated genes. (*N*) Motifs beneath Tbx2 ChIP peaks in Tbx2-activated versus Tbx2-repressed genes.

Our results so far suggest that Tbx2 controls positively genes implicated in promoting cell division and represses genes linked to cell cycle arrest. However, genes controlled by Tbx2 are not necessarily directly regulated by it. Therefore, to derive a robust list of genes bound and regulated by Tbx2, we integrated the results from the RNA-seq analysis with those from the ChIP-seq data set. The results enabled us to generate a robust list (Supplemental Table S3) of genes whose expression was up-regulated or down-regulated by Tbx2 depletion and that were also bound by Tbx2 in the ChIP-seq experiments. The overlap between the ChIP peaks and differentially expressed genes is shown in Supplemental Figure S5A. Examples of Tbx2-bound genes whose expression is changed on Tbx2 depletion is shown in Figure 5A, and the corresponding mapped ChIP-seq peaks are shown in Figure 5, B–K, or for *Pten* in Figure 3B. Notably, depletion of Tbx2 increased expression of *Cdkn1a* (p21), a cyclin-dependent kinase inhibitor implicated in p53-mediated senescence that has been shown previously to be bound and repressed by Tbx2 (Prince et al. 2004; Vance et al. 2005). *Cdkn1a* was bound at two distinct locations: at a consensus AGTGTGGA consensus T-element at the transcription start site (TSS) as described previously (Prince et al. 2004; Vance et al. 2005) but also at a second intronic site highly enriched in GTG motifs. We also confirmed that the *E2f1* gene was bound and regulated by Tbx2 (Fig. 5E) and that Tbx2 knockdown also affected expression of a number of additional E2f family members (Supplemental Fig. S5B), including *E2f3*, which was bound by Tbx2 close to the transcription start site (Supplemental Fig. S5C).

Analysis of genes bound and regulated by Tbx2 using the Kyoto Encyclopedia of Genes and Genomes (KEGG) (Fig. 5L) supports a conclusion that Tbx2 controls a broad gene expression program promoting DNA replication and the cell cycle. A full list of genes implicated in G1/S or G2M that are directly bound and regulated by TBX2 is in Supplemental Table S4. Notably, Hippo signaling was down-regulated by Tbx2 depletion, consistent with Tbx2 binding motifs bound by TEAD, which lies downstream from the Hippo pathway.

Given that Tbx2 appears to bind genes that are both activated and repressed by its depletion, we next asked whether the distribution of binding sites in the repressed or activated genes differed. The results (Fig. 5M) revealed only minor differences in distribution of Tbx2 binding sites between genes up-regulated or down-regulated on Tbx2 depletion. Remarkably, however, the motifs enriched beneath the ChIP peaks of Tbx2 up-regulated versus down-regulated genes were different (Fig. 5N). For example, T-element-related motifs were found in the Tbx2-repressed genes, whereas the CACGTG E-box motif was restricted to the up-regulated genes.

### TBX2 interacts with the BCOR/PRC1.1 complex

**Figure 6.**
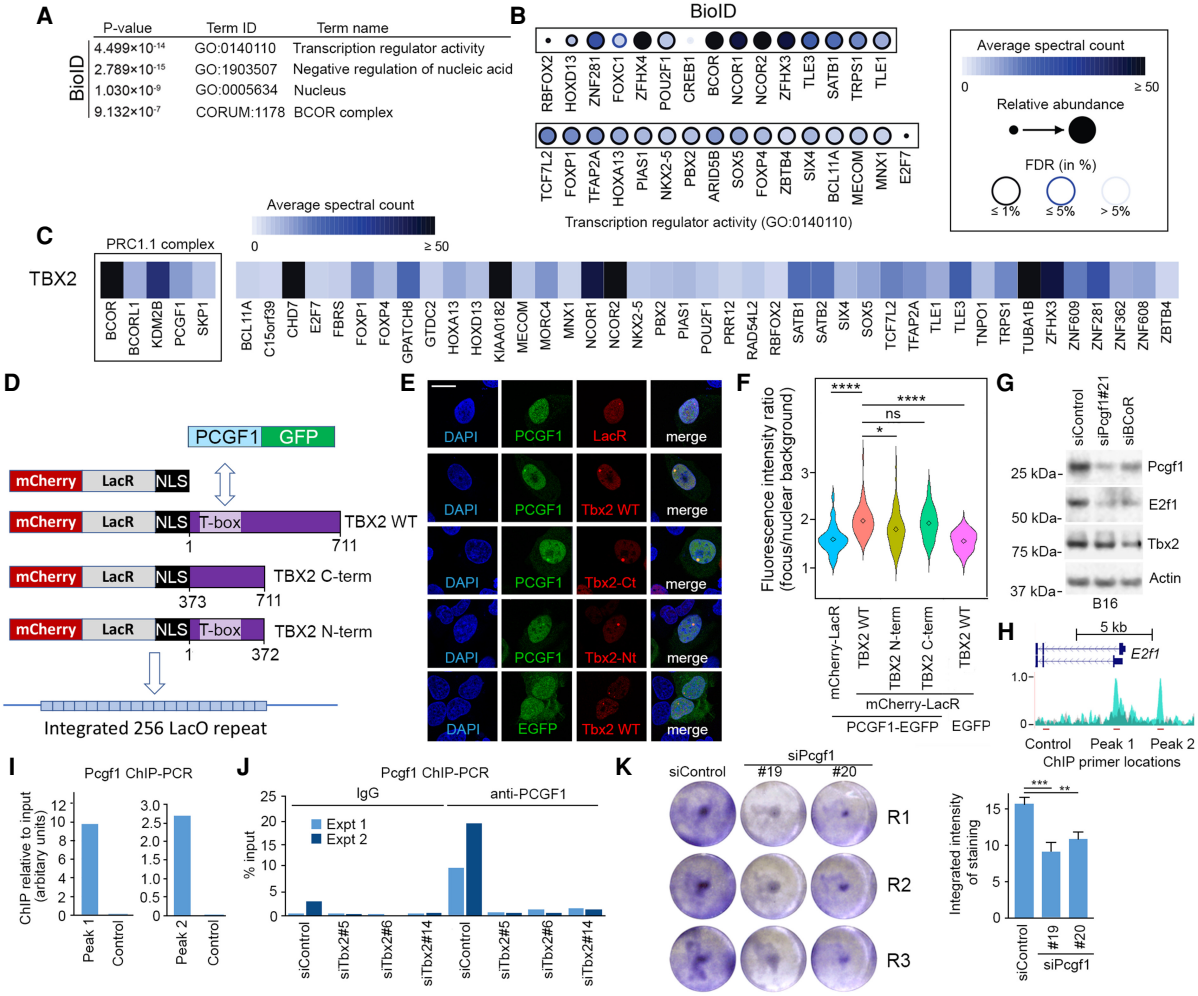
Tbx2 interacts with the BCOR/PRC1.1 complex. (*A*) GO analysis of Tbx2-interacting factors identified by mass spectrometry analysis. (*B*) Mass spectrometry BioID results showing Tbx2-interacting factors categorized by the GO term “transcription regulator activity.” Note that because Tbx2 can be autobiotinylated in the BioID analysis, it was removed from the list of proteins detected. (*C*) Tbx2-interacting factors identified, with BCOR/PRC1.1 complex components grouped to the *left*. (*D*) Nuclear tethering assay. (*E*) Representative images of cells transfected with the indicated expression constructs. Scale bar, 20 mm. (*F*) Quantification of nuclear tethering assay. Diamonds within plots indicate mean, and the width indicates probability density at a certain fluorescence intensity ratio. (ns) Not significant, (*) *P* < 0.05, (****) *P* < 0.0001, Student's *t*-test. (*G*) Western blot showing expression of the indicated proteins 48 h after transfection with two different siRNAs to deplete Pcgf1 or BCoR. (*H*) ChIP-seq showing locations of Tbx2 binding sites at the *E2F1* locus. (*I*) ChIP-qPCR using anti-Pcgf1 and primers specific for the indicated regions of the *E2f1* locus shown in *H*. Quantification of the PCR products is shown for Tbx2 peaks 1 and 2 and a control region not bound by Tbx2. (*J*) ChIP-qPCR using anti-Pcgf1 or IgG control at *E2f1* peak 1 performed 48 h after transfection of B16 cells with control or Tbx2-specific siRNAs. (*K*) Triplicate assay for B16 cell growth after transfection with control or two Pcgf1 siRNAs as indicated. Quantification was determined by integration of the staining intensity after subtraction of background. Error bars indicate SD. (**) *P* = 0.002, (***) *P* = 0.001 Student's *t*-test.

As a regulator of gene expression, TBX2 is expected to physically interact both with transcription factors to provide sequence specificity and flexibility in driving specific gene expression programs and with cofactors that may impose regulation on the genes bound by remodeling or modifying chromatin. To determine the repertoire of Tbx2-interacting factors, we stably expressed in HEK293 cells FLAG-tagged Tbx2 as a fusion with an abortive BirA* (R118G), allowing us to perform proximity biotinylation experiments (BioID). The expression of BirA*-Tbx2 allowed for biotinylation of the factors in its proximity that were subsequently purified using streptavidin beads and identified using mass spectrometry. BirA-FLAG-GFP or BirA coupled to a nuclear localization sequence was used as negative control to enable the identification of proteins statistically enriched for BirA*-Tbx2 BioID. This system was chosen to identify Tbx2-interacting factors since it covalently marks proteins of interest with biotin, allowing for the use of very harsh lysis conditions to solubilize most nuclear proteins prior to their isolation with streptavidin beads. A full list of interacting factors and a summary of the mass spectrometry data are in Supplemental Table S5. Gene ontology analysis of the mass spectrometry results obtained using a false discovery rate of 1% (Fig. 6A) revealed interaction of Tbx2 with factors implicated in gene regulation and nuclear functions, most notably including the BCOR/polycomb repressive complex 1.1 (PRC1.1) complex (*P* = 9.132 × 10^−7^) comprising BCOR, BCORL1, KDM2B, PCGF1, and SKP1, which can play both positive and negative regulatory roles in gene expression (Gil and O'Loghlen 2014; Cohen et al. 2019; Geng and Gao 2020). In addition to multiple components of the BCOR complex, other cofactors identified include the nuclear receptor corepressor 1 (NCOR1) and NCOR2 complexes (Mottis et al. 2013) and CHD7, a helicase implicated in chromatin remodeling (Bouazoune and Kingston 2012).

The ability of T-box factors like TBX2 to regulate their target genes requires that they bind DNA, but their low DNA binding specificity (Fig. 3F,G; Supplemental Fig. S3E) dictates that they need to cooperate with other sequence-specific transcription factors. Consistent with this, the mass spectrometry analysis (Fig. 6B,C) revealed a range of potentially cooperating transcription factors, including NKX2.5, a homeodomain transcription factor already known to facilitate cooperative DNA binding by TBX2 (Habets et al. 2002); TCF7L2 (TCF4), which recruits β-catenin to target genes and is preferentially expressed in invasive phenotype melanoma cells (Eichhoff et al. 2011); and TFAP2, which plays a key role in controlling melanocyte differentiation (Seberg et al. 2017b). Note that although 31% of the TBX2-bound sites identified by ChIP-seq contained a CACGTG E-box motif (Fig. 3D) recognized by MITF (and related family members TFEB and TFE3) and USF1/USF2, none of these factors was detected by our mass spectrometry analysis as interacting with TBX2. Note that both USF1 and USF2 as well as TFEB, although not MITF, are expressed in the HEK293 cells used for the mass spectrometry (Huan et al. 2005; Matsuda et al. 2013; Ploper et al. 2015).

Given the multiple members of the BCOR/PRC1.1 complex that we identified as interacting with TBX2, we sought to confirm the interaction using an orthogonal approach. Specifically, we determined whether Tbx2 could interact with the PCGF1 subunit of the BCOR/PRC1.1 complex by using a live-cell nuclear tethering assay (Fig. 6D). To this end, Tbx2 was expressed as a fusion protein with the Lac repressor and mCherry, enabling visualization in live cells of a red fluorescent focus within nuclei corresponding to Tbx2 tethered to an array of 256 repeats of the Lac operator integrated into the genome. If PCGF1 were able to interact with Tbx2, coexpression of an EGFP-PCGF1 fusion would result in colocalization of the green fluorescent PCGF1 protein with the red focus corresponding to Tbx2. As controls, we used an mCherry-LacR vector without Tbx2, as well as deletion mutants lacking the N-terminal (amino acids 373–711) or C-terminal (amino acids 11–372) regions of Tbx2 (Fig. 6D). Example results are shown in Figure 6E, with quantification shown in Figure 6F. These reveal colocalization of PCGF1-EGFP with mCherry-LacR-Tbx2, and only background colocalization with the mCherry-LacR control. Colocalization with mCherry-LacR-Tbx2 C-terminal region was similar to Tbx2 WT, and was moderately reduced using the N-terminal domain containing the T-box. These data confirm that Tbx2 can interact in live cells with PCGF1. In similar assays using USF1 and USF2 fused to GFP (data not shown), we saw no consistent localization with mCherry-LacR-Tbx2 above that seen with the controls. Consistent with these observations, depletion of Pcgf1, or the BCoR component of the PRC1.1 complex, in B16 cells led to reduced E2f1 expression (Fig. 6G), suggesting it is the PRC1.1 complex rather an Pcgf1 alone that is responsible for regulation of E2f1 expression. The reduction in E2f1 expression following Pcgf1 depletion in B16 cells was reproduced using two additional Pcgf1-specific siRNAs (Supplemental Fig. S6A), although siPcgf1#19 was less efficient at reducing E2f1 than siPcgf1#20. The reduced ability of siPcgf1#19 to decrease E2f1 expression, despite efficiently silencing Pcgf1, might be because it caused a moderate increase in Tbx2 levels, unlike siPcgf1#20 (Supplemental Fig. S6B), siPcgf1#21 (Fig. 6G), or siRNA targeting human PCGF1 in 501mel or A375M cells (Supplemental Fig. S6C). ChIP analysis indicated that Pcgf1 bound both Tbx2-binding sites in the *E2f1* promoter, but not at a control region within the *E2f1* gene body (Fig. 6H,I). Importantly, in two independent experiments, knockdown of Tbx2 using three different siRNAs reduced the occupancy of Pcgf1 at the *E2f1* locus using IgG as a negative control (Fig. 6J), confirming that Tbx2 is necessary for Pcgf1 recruitment, while knockdown of Pcgf1, like E2F1 knockdown (Fig. 4F), led to decreased cell growth (Fig. 6K).

## Discussion

In development and in the wide range of cancers where it is overexpressed, Tbx2 has been implicated in proliferation, senescence bypass, and cell invasion (Abrahams et al. 2010; Wansleben et al. 2014; Decaesteker et al. 2018). However, mechanistically, how it exerts its biological effects has been poorly defined. Previous work to identify direct TBX2 target genes by ChIP-seq used an antibody against the endogenous protein. For example, using neuroblastoma cells, Decaesteker et al. (2018) reported 557 TBX2 binding sites (*q* < 0.05) in total (41% intergenic; 30% lincRNAs; binding to consensus AGGTGTGA), while a more recent study of embryonic lungs (Lüdtke et al. 2021) identified a total of 3062 Tbx2 peaks enriched >3.5-fold over the control, but only 177 were found within 5 kb of transcription start sites (TSSs), and in neither study were replicates used to robustly identify target sites. In contrast, by using high-affinity anti-HA antibody and HA-tagged endogenous Tbx2 in melanoma, we were able to identify up to 7500 binding sites (clone 9, R2) with a robust set of sites identified in common between multiple replicates, of which ∼38% were within 3 kb of the transcription start site. These are likely to represent bone fide Tbx2 recognition sites given that we used multiple replicates and identified binding to a number of genes such as *CDH1*, *PTEN*, and *NDRG1* that had been noted previously as being TBX2-regulated. Moreover, we also revealed not only binding to a consensus T-element, but also recognition of CACGTG-type E-boxes as well as SOX10 and TEAD motifs, each of which contains a core GTG sequence in common with the T-element. This result was surprising, but is likely to be explained by the unusual mode of DNA recognition by T-box factors.

In vitro, both a T-element and an E-box were recognized with by a bacterially expressed and purified Tbx2 T-box DNA-binding domain with a K_D_ of ∼602 nM and 86 nM, respectively. For a transcription factor, this is relatively weak, especially for the T-element. For example, fluorescence anisotropy indicated that the microphthalmia-associated transcription factor MITF binds the same E-box with a K_D_ of ∼40 nM (Louphrasitthiphol et al. 2020), although recent measurements using an electrophoretic mobility shift assay suggest that DNA binding by MYC/MAX to an E-box is on the order of 3.78 μM (Pellanda et al. 2021). Moreover, we also showed binding in vitro to SOX, TEAD, and GATA sequences with an affinity similar to that of the E-box. One likely reason for the relatively nonspecific DNA binding by Tbx2 in vitro is that T-box factors make base-specific contacts with only two G residues and an α-helix inserted into the minor groove (Müller and Herrmann 1997; Coll et al. 2002; El Omari et al. 2012). Since the minor groove in standard B-form DNA is too narrow to accommodate an α-helix, the minor groove must be widened to enable T-box factor DNA recognition. This might occur either through DNA distortion mediated by cooperating DNA-binding factors or on the surface of a nucleosome where the wrapping of DNA around the histone core will generate widened major and minor grooves. Consistent with this, TBX2 can bind nucleosomal DNA and indeed can recognize DNA across two gyres of the DNA wrapped around the histone octamer core (Demay et al. 2007; Zhu et al. 2018). Therefore, it seems likely that while a T-element may be selected as a binding site, in an in vitro fluorescent anisotropy experiment the minor groove is not readily recognized by the Tbx2 DNA binding domain. Nevertheless, the sequences identified beneath the peaks in our ChIP-seq analysis are highly significant (*P* = < 0.01) and reproducible. Although we cannot rule out the possibility that binding at some sites may occur through indirect long-range interactions such as have been observed previously for insulator binding proteins (Liang et al. 2014), we feel it more likely that DNA recognition in vivo by Tbx2 and other T-box transcription factors will be dictated by the chromatin landscape combined with binding of cofactors that facilitate Tbx2 DNA recognition. Our data therefore suggest that the consensus T-element is unlikely to represent a unique recognition motif for Tbx2 or other T-box factors, but rather T-box factors will bind to a range of sites dictated by the presence of appropriate DNA topography and DNA-binding cofactors. In this respect, our mass spectrometry analysis identified a wide range of transcription factors able to interact with TBX2, many of which we anticipate may contribute to TBX2 DNA binding in vivo.

Remarkably, while the genomic distribution of Tbx2 binding did not differ between genes activated or repressed on Tbx2 depletion, the sequences bound by Tbx2 were different. For example, the classical T-element was restricted to genes up-regulated on Tbx2 depletion, while the E-box was found in the down-regulated genes. This observation suggests that Tbx2's capacity to regulate gene expression may depend on cooperation with additional transcription factors that facilitate recognition of different sequence elements. One interesting possibility is that by binding in the minor groove, Tbx2 can cooperate with other transcription factors binding in the major groove; for example, basic helix–loop–helix factors that recognize CACGTG E-box motifs. This would potentially include MITF, a key regulator of melanocyte and melanoma biology (Goding and Arnheiter 2019), or USF1/2. However, MITF is not expressed in many Tbx2-expressing cell types, and we failed to detect interaction with USF1 or USF2 using a range of assays. However, E-boxes, as well as the other motifs bound by Tbx2, can be recognized by additional factors, and further work will be required to identify the determinants of Tbx2 DNA binding specificity in vivo.

**Figure 7.**
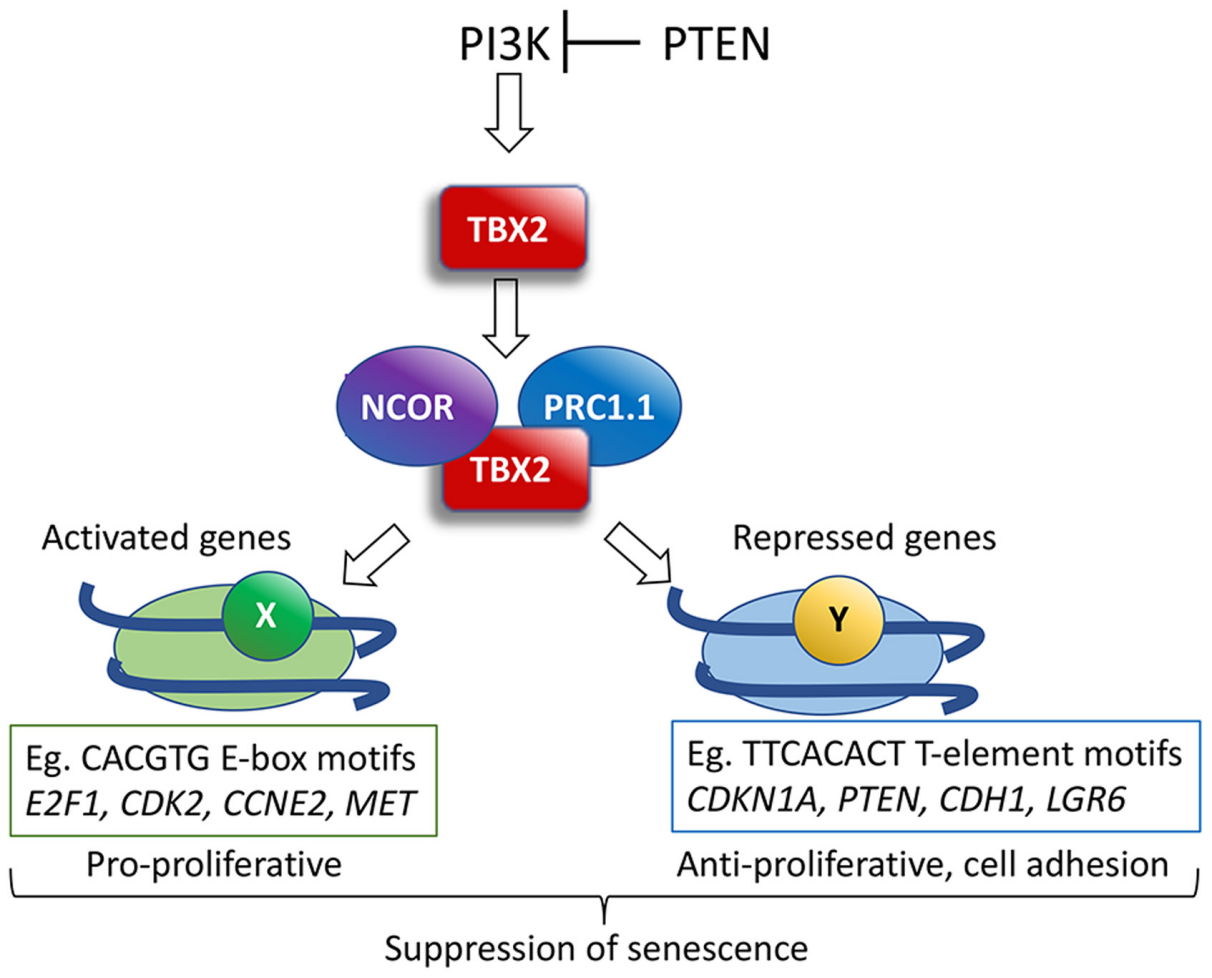
Summary of TBX2 function. TBX2 expression is activated by PI3K signaling, including via loss of *PTEN*, and interacts with chromatin remodeling complexes, including PRC1.1 and the NCOR complex, although whether different interacting complexes regulate different genes or interact with TBX2 at different times is unclear. Note that while multiple components of the PRC1.1 complex and NCOR were detected using mass spectrometry, only interaction with Pcgf1 was validated using the nuclear tethering assay. TBX2 will recognize nucleosomal DNA at loci using DNA-binding cofactors to dictate binding specificity, with genes repressed or activated after Tbx2 depletion exhibiting different sequences associated with TBX2 binding. Activated genes tend to be proproliferative and repressed genes tend to be antiproliferative or antimigration, and the resulting gene expression program driven by TBX2 will suppress senescence. While TBX2 has been characterized as a repressor, it is possible its capacity to activate or repress transcription is mediated by the cooperating transcription factors.

Examining the repertoire of Tbx2-bound and -regulated genes indicated that Tbx2 plays a broad role in gene regulation implicated primarily in cell cycle progression, and, as shown here and elsewhere, depletion of Tbx2 leads to cell cycle arrest and senescence. Consistent with this, previous work had established that TBX2 is a transcriptional repressor that could suppress expression of the senescence-associated Cyclin-dependent kinase inhibitors (CDKis) *CDKN1A* (p21) and *CDKN2A* (p16^INK4a^), and consequently loss of TBX2 would lead to decreased proproliferative gene expression programs. Our results provide a fundamentally different perspective on Tbx2 function in senescence and development, revealing that rather than acting as a dedicated transcriptional repressor, many directly bound genes are potentially activated by Tbx2, including that encoding E2f1, a proproliferative and known antisenescence transcription factor (Rowland et al. 2002). Notably, gene ontology analysis suggests that in addition to repressing genes that block the cell cycle, Tbx2 maintains expression of genes associated with promoting cell cycle progression, such as those regulated by Myc, as well as genes implicated in DNA damage repair. Consistent with Tbx2 promoting cell cycle progression, *Cdk2* mRNA expression was down-regulated on Tbx2 depletion, and Tbx2 bound to a *Cdk2* intron. Similarly, the *E2f1*, *Ccne2* (Cyclin E), and *Met* genes, all of which promote passage through S phase, are revealed as directly Tbx2-bound genes that are down-regulated on Tbx2 depletion. The proproliferative role for Tbx2 revealed by integrating the ChIP and RNA-seq data reinforces the outcome from the GSEA of human tumors. Although further work is necessary to confirm the biological significance of the regulation of the target genes by Tbx2, our conclusion is that rather than suppressing senescence by regulating a single gene, the role of Tbx2 is to implement a broad-ranging antisenescence and proproliferative gene expression program (Fig. 7). However, as many of the apparently Tbx2-activated genes also contain E2F binding sites and Tbx2 regulates expression of E2F family members, some effects on gene expression following depletion of Tbx2 could arise indirectly via decreased E2f1. Notably, whether Tbx2 is a bone fide transcription activator in some contexts awaits confirmation that may be difficult to obtain. For example, Tbx2 binds nucleosomal DNA where precise nucleosome positioning is required for target sequences to be accessible. However, fusion of regulatory sequences to reporters can lead to aberrant nucleosomal positioning across a promoter with as little as a 2-bp shift in wrapping of DNA around the histone octamer, leading to fundamental changes in requirements for gene regulation (Martinez-Campa et al. 2004). Moreover, mutation of a Tbx2 binding site in a genomic context (an E-box, for example) may disrupt binding of transcription factors able to recognize the same sequence. Nevertheless, our results are currently consistent with Tbx2 being required both to maintain and repress transcription.

The ability of TBX2 to bind and activate or repress transcription will be dependent on a wide range of factors. These include the location of its binding sites in relation to other regulatory elements and the regional epigenetic landscape, as well as the identification of its interacting cofactors that may facilitate targeting to specific loci and its capacity to remodel chromatin. Moreover, cooperating transcription factors whose binding is enhanced by interaction with Tbx2 could themselves mediate transcription activation, rather than Tbx2 itself. To date, in addition to the homeodomain transcription factor NKX2.5 (Habets et al. 2002), TBX2 has been identified as interacting with HDAC1 (Vance et al. 2005), RB1 (Vance et al. 2010), a range of transcription factors such as HMGB2 and PBX1 identified by mass spectrometry (Lüdtke et al. 2021), EGR1 (Redmond et al. 2010), PML (Martin et al. 2012), and the heterochromatin proteins CBX (Lüdtke et al. 2021) and HP1α (Crawford et al. 2019). Tbx2 also was reported to bind components of the NuRD chromatin remodeling complex (Lüdtke et al. 2021). Our mass spectrometry analysis identified many of these factors (e.g., HDAC1, HDAC2, CHD4, PBX1, and NKX2.5) but below our stringent 1% FDR statistical cutoff. Nevertheless, confirmation of HDAC1/2 interaction with TBX2 in proximity ligation assays (Lüdtke et al. 2021) suggests that even the lower-stringency interactors detected here may be relevant for Tbx2 function. Note that one limitation of our mass spectrometry analysis was that it was performed in HEK293 cells rather than in melanoma cells, meaning that we may have missed interaction with some melanocyte/melanoma-specific factors. Nevertheless, as TBX2 is widely expressed in many cell types, the interacting factors identified here are likely to be relevant for TBX2 function.

Beyond the wide range of Tbx2-interacting transcription factors detected, which presumably act to facilitate cooperative recruitment to, and regulation of, target genes, Tbx2 also interacts with a number of chromatin remodeling factors, including CHD7, BCL11A, NCOR1, and NCOR2. In addition, we found Tbx2 interaction with BCOR that participates in the formation of the PRC1.1 complex, one of six noncanonical PRC1 complexes described to date (Piunti and Shilatifard 2021). We also found Tbx2 associated with additional BCOR/PRC1.1 components BCORL1, PCGF1, KDM2B, and SKP1, while RING1 and USP7 were identified but fell below our 1% FDR statistical cutoff. Although we did not detect interaction with the PRC1.1 complex factors RYBP or YAF2, this may be because their orientation within the PCR1.1 complex would not permit their biotinylation and purification using the BioID approach. We confirmed the interaction between Tbx2 and PCGF1 using a nuclear tethering assay and Pcgf1 was also found by ChIP at the Tbx2 binding sites in the *E2f1* gene, where its recruitment was dependent on Tbx2 expression, and, like Tbx2, it was required for E2f1 expression. Like TBX2, PRC1 is implicated in maintenance of cell identity, choice of cell fate, and transitions between cell states associated with specific lineages (Vidal and Starowicz 2017). More specifically, PCGF1 is required for proliferation, and in a zebrafish knockout model, loss of PCGF1 leads to premature aging characterized by increased senescence (Dupret et al. 2016). Similar observations have been made for KDM2B, which protects mouse embryonic fibroblasts from senescence by repressing the *CDKN2a* (p16^INK4a^) gene (Tzatsos et al. 2009), as does Tbx2 (Jacobs et al. 2000). However, while some Tbx2 functions may be regulated by interaction with the BCOR/PRC1.1 complex, the association with Tbx2 of multiple epigenetic regulators identified here and in other studies (Vance et al. 2005; Lüdtke et al. 2021) suggests that Tbx2 may use different cofactors depending on context. For example, different cofactors may be used at different times in the cell cycle (Bilican and Goding 2006), in response to different signaling pathways regulating Tbx2 function, or depending on its DNA-binding cofactors at specific binding sites.

Given the proproliferative and antisenescence program driven by TBX2, understanding how its expression is regulated is also a key issue. Previous work identified *TBX2* as a target for retinoic acid receptor (Boskovic and Niles 2004), a PML–E2F4 complex (Martin et al. 2012), Sonic Hedgehog (Lüdtke et al. 2016), and WNT (Aydoğdu et al. 2018) as well as PAX3, a lineage-restricted transcription factor that plays a key role in melanocyte development and in melanoma (Liu et al. 2013). Here we significantly extended these observations to reveal that *TBX2* is regulated by PI3K signaling. This is important given the critical role of the PI3K pathway in promoting senescence bypass in fibroblasts (Kennedy et al. 2011), as well as in melanoma, where loss of *PTEN* can cooperate with activated *BRAF* or *NRAS* to promote tumor initiation and metastatic dissemination (Nogueira et al. 2010; Vredeveld et al. 2012). We show that TBX2 expression is up-regulated in human tumors and mouse models where *PTEN*, a negative regulator of PI3K signaling, is inactivated. The control of TBX2 by PI3K therefore makes biological sense, and is reflected in the ontology of the TBX2-regulated genes identified here. Therefore, it seems likely that the effects of PI3K signaling on melanoma growth, progression, and senescence bypass may be mediated at least in part by TBX2. Significantly, we also reveal that TBX2 directly represses *PTEN* in melanoma, a result consistent with observations in rhabdomysosarcoma (Zhu et al. 2016) and nasopharyngeal carcinoma (Lv et al. 2017), where TBX2-mediated repression of *PTEN* mRNA expression has been observed. Since PTEN represses PI3K, and TBX2 is activated by PI3K signaling, our results suggest TBX2 participates in a positive feedback loop, a mechanism used biologically to stabilize specific cell states. Finally, we also note that in the absence of *Pten* loss, the genetically defined mouse tumors with activating *Braf* mutations exhibited a lower expression of *Tbx2* than the *Nras* mutant tumors, presumably because Nras can signal via PI3K. However this difference was not reproduced in human tumors where the median *TBX2* expression was not significantly different between tumors with activated *NRAS* or *BRAF*. The difference between mouse and human tumors may reflect the fact that human tumors are more genetically heterogenous or that the microenvironment (for example, signaling human tumors) may exhibit a significant amount of immune cell infiltration that is usually reduced in mouse tumors. Nevertheless, our results do provide compelling evidence that TBX2 is up-regulated by PI3K signaling that is associated with melanoma progression.

## Materials and methods

### Cell lines

All melanoma cell lines were authenticated by STR analysis using Eurofins Genomic service. All parental and derivative cell lines were verified mycoplasma-free using Ludwig Cancer Research monthly mycoplasma testing service. Cells were grown in RPMI media supplemented with 10% fetal bovine serum (FBS) without antibiotics. When reaching confluence, cells were passaged 1:6 to 1:10 depending on cell line using trypsin. Tagging of endogenous Tbx2 in B16 melanoma cells was performed using 3xHA and was carried out according to the protocol of Stewart-Ornstein and Lahav (2016).

### Bacterial strains

*Escherichia coli* DH5α bacteria genotype F^−^ Φ80*lac*ZΔM15 Δ(*lac*ZYA-*arg*F) U169 *rec*A1 *end*A1 *hsd*R17(r_k_^−^, m_k_^+^) *pho*A *sup*E44 *thi*-1 *gyr*A96 *rel*A1 λ^−^ (Invitrogen 18265017) was used.

### Plasmids

The px330 plasmid was a gift from Maike Effern (Bonn, Germany). peFLUT1a-3xHA-NeoR was constructed by inserting the 3xHA tag into the XhoI and EcoRI sites in the peFLUT1a-NeoR vector, which was a gift from Dr. Vincenzo D'Angiolella. peGFP-USF2 plasmid was constructed by inserting USF2 CDS into the XhoI and EcoRI sites in pEGFP-C1 plasmid. The mCherry-LacR-plasmid was provided by Dr Nico Dantuma (Stockholm, Sweden). TBX2 WT or deletion mutants were cloned into the BamHI site of the mCherry-LacR plasmid.

### eFlut tagging of the endogenous Tbx2 gene

To direct Cas9 to cut the C terminus of the endogenous *Tbx2* gene, single-guide RNA (sgRNA) was designed to target the following sequence near the stop codon: GCCGGGAGTCGCCCAAGTGAGGG. To clone the sgRNA into the px330 plasmid, the top (5′ → 3′) CACCGGCCGGGAGTCGCCCAAGTGA and bottom strand (5′ → 3′) AAACTCACTTGGGCGACTCCCGGCC were annealed and then cloned into the BbsI site of px330 (px330-SL007). To design the primers for PCR amplification of the donor template with homology arms, the 40 bp immediately preceding the stop codon and the 40 bp immediately after were selected. Then, 20 bp from the N terminus of the 3xHA tag were added to the forward primer, and 20 bp from the C terminus of the selection marker (the neomycin resistant gene) were added to the reverse primer. To eliminate the sgRNA site, the PAM site was removed from the donor sequence and a silent mutation was introduced to the last base before the PAM. For tagging the C terminus of Tbx2 with 3xHA, forward primer SL095 and reverse primer SL069 were used to amplify the donor template from peFLUT1a-3xHA-NeoR. The primers designed above and respective template plasmids were used to make donor sequences for homology-directed repair (HDR) using Phusion polymerase with HF buffer.

Thirty-thousand B16 cells were plated in 2 mL of growth media per well in a six-well plate. *Trans*-IT-LT1 reagent was warmed to room temperature and 200 μL of Opti-MEM serum-free media was placed in a sterile tube. One microgram of Cas9-sgRNA plasmid (px330-SL007) and 1 μg of homology donor were added to the medium and mixed. Ten microliters of LT1 reagent was added to the diluted DNA mixture (1:5 ratio of DNA:lipid mix) and gently mixed and the mix incubated for 15 min at room temperature before the DNA lipid mix was then added dropwise to the cells. Forty-eight hours after transfection, cells were trypsinized and plated in a 10-cm dish. Cells were allowed to recover overnight from transfection and to express Cas9. B16 cells transfected with 3xHA-NeoR donor sequence were then selected in growth media containing 500 μg/mL G418 for 6 d until negative control cells, which were transfected only with the Cas9 plasmid but not the donor sequence, were eliminated. Viable cells were recovered in normal growth media for 3 d until individual clones were visible by eye. Primers flanking the coding region of Tbx2 C terminus and 3′ untranslated region (UTR) were used to generate a band that was distinguishable. The PCR reaction was designed so that a large band was apparent if the insertion was successful, a small band was apparent if not, and both bands were apparent if the clone was heterozygous or a mixed population. For B16-Tbx2-3xHA-NeoR clones, primers SL127/128 were used for the genomic DNA PCR. Then, nested PCRs were conducted (primers SL059/128 were used to amplify the 3xHA-NeoR-3′ UTR region, and primers SL127/132 were used to amplify the Tbx2-3xHANeoR region) followed by sequencing to confirm the locus was modified correctly.

### Western blotting

Cells were washed once with 1× PBS, and then lysed with RIPA buffer (150 mM NaCl, 50 mM Tris, 0.1% [w/v] SDS, 0.5% [w/v] sodium deoxycholate, 1% Triton X-100). Plates were rotated for 10 min at 4°C, and then cell lysates collected and transferred to Eppendorf tubes. We added 2× Laemmli buffer (125 mM Tris-HCl, 4% [w/v] SDS, 1% bromophenol blue, 10% β-mercaptoethanol, 20% glycerol) to the RIPA buffer, and the mixture was placed in a heat block for 15 min at 95°C. Whole-cell protein lysates were subjected to 10% polyacrylamide SDS- PAGE before proteins were transferred onto nitrocellulose membranes (Amersham Biosciences). Membranes were blocked with 5% nonfat milk in PBS containing 0.1% Tween 20 and probed with the appropriate primary antibodies overnight at 4°C. Proteins were detected using antimouse or antirabbit immunoglobulin coupled to horseradish peroxidase (Bio-Rad and Santa Cruz Biotechnology) and visualized with an ECL detection kit (GE RPN2106).

### Pathology

The antibody used for immunohistochemistry corresponding to human melanoma tissue was rabbit anti-Tbx2 HPA008586 (Sigma-Aldrich): low-expressing sample: male, aged 41, metastasis; high expressing sample: female, aged 82, metastasis. Images obtained from the Human Protein Atlas (https://www.proteinatlas.org/ENSG00000121068-TBX2/pathology/melanoma).

### Mouse melanoma tumors

Mice were bred and maintained in the specific pathogen-free mouse colony of the Institut Curie, in accordance with the institute's regulations and French and European Union laws. The generation of transgenic mice and associated melanoma were described previously (Lesche et al. 2002; Delmas et al. 2003; Ackermann et al. 2005; Yajima et al. 2006; Dhomen et al. 2009; Conde-Perez et al. 2015).

### Ethical rules

Animal care, use, and experimental procedures were conducted in accordance with recommendations of the European Community (86/609/EEC) and Union (2010/63/UE) and the French National Committee (87/848). Animal care and use were approved by the ethics committee of the Curie Institute in compliance with the institutional guidelines. Experimental procedures were specifically approved by the ethics committee of the Institut Curie CEEA-IC 118 (CEEA-IC 2016-001) in compliance with the international guidelines.

### Immunofluorescence

Adherent cells were grown on glass coverslips in 12-well plates until 80% confluence. Growth media was removed and cells were washed once with 1× PBS. PBS was then removed and 4% paraformaldehyde (PFA) was added, and cells were fixed for 10 min at room temperature. PFA was removed, and cells were washed twice with PBS before being permeabilized and in blocking solution (5% BSA in PBS + 0.1% Triton X-100) for 20 min at room temperature. Primary antibodies were diluted in blocking solution, added to wells, and incubated for 1 h at room temperature. Primary antibodies were then removed, cells were washed twice with PBS, and secondary antibodies (conjugated to fluorescent labels) were diluted 1:1000 in blocking solution; 0.5 μg/mL DAPI was added. The mix was next incubated for 30 min at room temperature. Antibody DAPI mix was removed, cells were washed three times with PBS, and coverslips were mounted on polysine microscopy slides with 2 μL of VectaShield mounting medium (Vector Laboratories H-1000-10). Coverslip edges were sealed using nail polish. Samples were imaged using a Zeiss 710 microscope with 20× objective.

### Nuclear tethering assay

Twenty-four-thousand U2OS-LacO cells were plated in 24-well plates. In total, 250 ng of the bait LacR-mCherry plasmid and the GFP-tagged prey plasmids were diluted in 80 μL of Opti-MEM and 2 μL of FuGENE 6 added before mixing, incubation for 15 min at room temperature, and addition to cells. Twenty-four hours later, cells were passaged 1:6, transferred to an eight-well chambered coverglass (Nunc Lab-Tek, 155411), and allowed to attach overnight. Medium was then removed from the eight-well chamber, and cells were washed once with PBS before fixing with 4% PFA for 10 min at room temperature. PFA was then removed and cells were washed twice with PBS. DAPI (1 μg/mL) diluted in PBS was added to the well and incubated for 5 min at room temperature before the DAPI solution was removed and cells were washed three times with PBS. Chamber wells were mounted with 10 μL of VectaShield mounting medium (Vector Laboratories H-1000-10) and imaged using a Zeiss 710 with a 64× oil objective at 1280 × 740 resolution. Cells transfected with both plasmids were selected for imaging and quantification. To quantify the image data generated, DAPI staining was used to mask the nuclear area and nuclear area size (ns) was measured. mCherry staining was used to mask the nuclear focus (dot) area and the dot area size (ds) was measured. Average GFP intensity in the nuclear and dot area was measured (GFP*_n_* and GFP*_d_*) as well as total GFP total fluorescence in nuclear and dot area (GFP*_tn_* and GFP*_td_*). GFP dot/nucleus ratio was calculated as ratio = GFP*_d_*/(GFP*_tn_* − GFP*_td_*) × (*ns* − *ds*).

### Crystal violet staining

Twenty-thousand B16 cells were plated in a 12-well plate (0.88 mL of cell suspension) and transfected with 10 nM siRNA (diluted in 120 μL of Opti-MEM containing 3 μL of Lipofectamine RNAiMAX). After 24 h, the 1 mL of transfection mix was replaced with 0.88 mL of normal growth media and, after a further 24 h, cells were again transfected with 10 nM siRNA. After 48 h, the transfection mix was removed and cells were fixed with 2% PFA for 10 min. Cells were stained with 0.1% crystal violet for 10 min, washed, air-dried, and scanned using a Fuji FLA-5100 imager.

### Cell cycle analysis by flow cytometry

Cells were plated in six-well plates and transfected with 20 nM siRNA for 48 h. Cells were washed and trypsinized, and cell suspension was transferred to Eppendorf tubes on ice and centrifuged at 800*g* for 2 min at 4°C. The supernatant was then discarded and cells were resuspended in 300 μL of PBS. Pure ethanol (750 µL) was added and mixed immediately by pipetting. Cells were then incubated with ethanol for 1 h on ice before centrifugation at 800*g* for 2 min at 4°C. The supernatant was discarded, and cells were resuspended in 250 μL of PI staining solution and then incubated with propidium iodide staining solution for 40 min at 37°C. PBS (750 μL) was added and mixed, and then cells were centrifuged at 800*g* for 2 min at room temperature. The supernatant was discarded, and cells were resuspended in 300 μL of SS5 solution, and then analyzed in a FACS Fortessa machine.

### Chromatin immunoprecipitation sequencing and qPCR (ChIP-seq and ChIP-qPCR)

B16-Tbx2-3xHA-NeoR cl#02 and cl#09 cells were cultured in 15-cm dishes using RPMI-1640 media. Fifteen 15-cm dishes were used for each replicate of ChIP-seq, and three were used for ChIP-qPCR. Cells were cross-linked for 10 min by adding formaldehyde (Sigma F8775) to a final concentration of 0.8%, and then quenched for 10 min by adding glycine to a final concentration of 200 mM. Cells from three 15-cm dishes were then washed, scraped, and collected into a 50-mL falcon tube (Corning 430828), and centrifuged at 1500*g* for 10 min. Cell pellet was lysed in 1 mL of ChIP lysis buffer (50 mM Tris-HCl at pH 8.0, 10 mM EDTA, 10 mM sodium butyrate, 1% SDS, 4× PIC [Roche 05056489001]) and passed through a 100-μm cell strainer before being sonicated for 15 min in a Covaris S220 (peak incident = 145 W, duty factor = 5%, cycle/burst = 200) until 200- to 400-bp fragments were obtained (assessed by 1% agarose gel). The sonicated chromatin was cleared by centrifugation at 13,000*g* for 10 min and the supernatant was diluted eightfold in ChIP dilution buffer (16.7 mM Tris at pH 8.0, 167 mM NaCl, 1.2 mM EDTA, 1% Triton X-100, 0.01% SDS) before 12 μg of anti-HA antibody (Roche 11666606001) or anti-Pcgf1 antibody (from Blackledge et al. 2014) was added, and chromatin was rotated in a 50-mL falcon tube overnight. In parallel, 60 μL of Dynabeads protein G (Invitrogen, 10004D) was washed, resuspended in ChIP dilution buffer, and blocked in 0.5 mg/mL BSA overnight at 4°C. Immunoprecipitation was carried out using blocked Dynabeads for 1 h at 4°C. The beads were washed three times each in low-salt wash buffer (20 mM Tris-HCl at pH 8.0, 150 mM NaCl, 2 mM EDTA, 1% Triton X-100, 0.1% SDS), high-salt wash buffer (20 mM Tris-HCl at pH 8.0, 500 mM NaCl, 2 mM EDTA, 1% Triton X-100, 0.1% SDS), and LiCl wash buffer (10 mM Tris-HCl at pH 8.0, 250 mM LiCl, 1 mM EDTA, 1% sodium deoxycholate, 1% NP-40) with beads transferred to a new DNA LoBind tube (Eppendorf Z666548) with each wash. The beads were eluted in 200 µL of elution buffer (100 mM NaHCO_3_, 1% SDS). Reversal of cross-linking of chromatin-immunoprecipitated DNA was done overnight at 55°C with addition of NaCl (final concentration 300 mM), 20 µg of RNase A, and 20 µg of Proteinase. Chromatin-immunoprecipitated DNA was recovered using a QIAquick PCR purification kit (Qiagen 28106). The concentration of chromatin-immunoprecipitated DNA used in sequencing was assessed using Qubit dsDNA HS assay kit (Invitrogen Q32851). Samples were subjected to 150-bp paired-end sequencing using a NovaSeq6000 (Illumina) at the Wellcome Trust Genomic Service, Oxford. qPCR reactions were performed with Brilliant II SYBR Green qPCR master mix (Agilent 600828) and analyzed using a Rotor-Gene Q. Melting curve analyses were carried out to ensure product specificity, and, to calculate the relative quantity of gene expression, a standard curve method was performed. Relative abundance of chromatin-immunoprecipitated DNA was normalized to 1% of input DNA. Three biological replicates were included in each experiment, and the data were represented as mean ± SD.

### RNA-seq of mouse melanoma cells in vitro

B16 and B16-Tbx2-3xHA-NeoR cl#02 and cl#09 (1.1 × 10^5^ of each) were plated in six-well plates (2.2 mL/well). siCN and siTbx2 were diluted in 150 μL of Opti-MEM (final concentration 20 nM). Lipofectamine RNAiMAX (7.5 μL; Invitrogen 13778030) was diluted in 150 μL of Opti-MEM (final concentration 0.3%). Diluted siRNA and diluted Lipofectamine were mixed together and incubated for 5 min at room temperature. Three-hundred microliters of siRNA–lipid mix was added dropwise to cells and mixed. At 24 h, transfection media was replaced with normal growth media; cells were allowed 24 h to recover. At 48 h, cells were lysed to collect RNA using RNeasy minikit (Qiagen 74106) according to the manufacturer's protocol. Fifteen microliters of 200 ng/μL extracted RNA from each sample (biological triplicates for all experiments) was submitted to the Wellcome Trust Genomic Service, Oxford. ERCC ExFold RNA spike-in mix (Ambion) was added prior to library preparation using QuantSeq 3′ mRNA-seq library preparation kit using 500 ng of starting material to minimize the PCR amplification step. Samples prepared were sequenced on HiSeq4000 (Illumina).

### ChIP-seq analysis

Prior to mapping to the mouse reference genome (mm10) with Bowtie2 (v.2.3.5) (Langmead and Salzberg 2012), quality of the raw sequencing data was evaluated using FastQC (v.0.11.7), and adapter contamination was removed using CutAdapt (v.2.8) when necessary. Peak calling was performed using MACS2 (v.2.1.2) (Zhang et al. 2008) taking a *q*-value of 0.01 as threshold. Bowtie2-generated SAM files were compressed to BAM files, indexed using SAMtools (v.1.9), sequentially converted to bigWig files using USCStools (v.373), and uploaded to the UCSC genome browser for visualization. Input and ChIP bigWigs were overlayed in the same track for a more intuitive presentation for peak quality. HOMER (v.4.8) was used to perform known motif enrichment and de novo motif identification, taking given peak size as input as we have paired-end data. Tag density plots were generated using seqMINER (v.1.3.4) (Ye et al. 2011), and overlap of peaks were generated using BEDTools intersect function (Quinlan and Hall 2010). Peak distribution across genomic features and relative to TSSs was obtained from the R package ChIPseeker. Venn diagrams of overlapped peaks were generated from the R package ChIPpeakAnno.

### RNA-seq analysis

The output raw Fastq files were examined for quality using Fastqc (version 0.11.7) and mapped against mm10 genome using STAR (version 2.6.1d). Reads per gene from STAR output BAM files were counted using FeatureCounts (subread/1.6.2). Counts per gene and sample information were converted to DeSeqDataSet objects and sequentially used as input for differential gene expression analysis using the R package Deseq2 (version 1.28.1). The Deseq function filters out lowly expressed genes, calculates normalization size factors, estimates dispersion, applies negative binominal GLM fitting, and calculates Wald statistics to identify differentially expressed genes (DEGs). Genes with an adjusted *P*-value <0.05 were considered statistically significant DEGs. Heat maps of RNA-seq samples were generated from counted reads in DeSeqDataSet object using the R package ComplexHeatmap. Raw reads were centered and scaled around the mean and hierarchically clustered. PCAs were generated from log transformed read counts using the R package ggfortify to visualize inter- and intragroup variability arising from transfection, clones, and replicates. Gene set enrichment analysis (GSEA) was carried out using the R package fgsea. One-thousand permutations were conducted for each gene set. Data were preranked by a gene significance score π-value combining expression fold change and statistical significance. π-value of the i-th gene in a data set was calculated as π_i_ = ‐φ_i_ (−log_10_ ρ_i_), where φ is the fold change and ρ is the adjusted *P*-value (Xiao et al. 2014). Gene ontology (GO) was carried out at http://geneontology.org using statistically significant DEGs, and the top 15 pathways were visualized using the R package ggplot2. KEGG pathway analysis was carried out in the R package clusterProfiler using the enrichKEGG function, and the visualization of individual KEGG pathways was generated using the pathview package. Visualization of the GSEA, GO, and KEGG pathways was done in ggplot2.

### Cloning, bacterial protein expression, and purification of the TBX2 T-box

The TBX2 DNA-binding domain (amino acids 94–281) was cloned by using restriction sites NcoI and XhoI into pETM14 (EMBL) in-frame with an N-terminal His tag and the 3C protease site. It was expressed in the *Escherichia coli* BL21(DE3) codon RIL strain. Cultures were grown in the terrific broth medium at 37°C to an optical density of 1.0–1.2, induced with 0.5 mM isopropyl thiogalactose (IPTG) for 4 h at 24°C, pelleted by centrifugation (5000×*g* for 30 min), and resuspended in 50 mL of lysis buffer (50 mM HEPES/NaOH at pH 7.5, 300 mM NaCl) containing DNase I and EDTA-free protease inhibitors (Roche). Cells were lysed three times by using an emulsifier under a constant pressure of 10,000 psi, and cell debris was removed by centrifugation (21,000×*g* for 40 min), filtered through a 0.45-µm filter, and loaded onto the pre-equilibrated HisTrap HP 5 mL (GE healthcare). The column was washed with 50 mL of lysis buffer, followed by 30–50 mM imidazole washes and then eluted with 250 mM imidazole. The eluted fusion protein was cleaved using 3C protease in the overnight dialysis buffer (lysis buffer). The cleaved fusion protein was then passed through the same HisTrapHP 5 mL (equilibrated with lysis buffer) to get rid of the His-tagged 3C protease while the TBX2(94–281) protein was in the flowthrough. The flowthrough was then concentrated using the 30-kDa concentrator (Merck KGaA) and loaded onto the pre-equilibrated (buffer used 50 mM HEPES at pH 7.2, 150 mM NaCl) Superdex 75 column size exclusion chromatography column (GE healthcare). The purified protein was aliquoted and stored at −80°C or used for biophysical experiments. Every step of the purification was analyzed with SDS-PAGE before proceeding to the next.

### Fluorescence anisotropy

Fluorescence-labeled oligonucleotides were synthesized at Metabion (Planegg/Steinkirscheny). Sequences are provided in the Supplemental Material. The E-box was annealed with the reverse complementary unlabeled E-box oligonucleotide through incubation for 5 min at 95°C, followed by a passive cooling step to room temperature. The fluorescein-labeled T-box was subjected to a similar annealing step followed by passive cooling without the reverse complementary unlabeled oligonucleotide considering the palindromic nature of the T-box sequence. Increasing concentrations of TBX2(94–281) protein were incubated with the respective dsDNA oligonucleotides at a final concentration of 3.33 nM for 10 min at 25°C in 50 mM HEPES/NaOH (pH 7.3) and 200 mM NaCl. Fluorescence anisotropy was then measured using an Infinite M1000 plate reader (TECAN) using the excitation diode at 470 nm and detecting the emitted light at 530 nm.

### Mass spectrometry

#### Cloning and cell line generation

Constructs for the genes of interest were generated via Gateway cloning into pDEST BirA*-FLAG-pcDNA5-FRT-TO as previously described (Lambert et al. 2019). Details of all entry clones and destination vectors used in this study are available on request. Details of all entry clones and destination vectors used in this study are in Supplemental Table S1, A and B. Bait proteins of interest were stably expressed in T-REx Flp-In HEK293 as described by Lambert et al. (2014). Parental Flp-In T-REx HEK293 cells, as well as stable cells expressing BirA*-FLAG, fused either to a green fluorescent protein (GFP) or to a nuclear localization sequence (NLS) were used as negative controls for the BioID experiments and processed in parallel to the bait proteins. Empty Flp-In T-REx HEK293 cells expressing GFP or fused to FLAG tag were used as negative controls for AP-MS experiments and processed in parallel to the bait-expressing cell lines. Stable cell lines were selectively grown in the presence of 200 μg/mL hygromycin up to 80% confluence before expression was induced via 1 μg/mL tetracycline and 50 μM biotin for 24 h and the cells were harvested. Cells were pelleted at low speed, washed with ice-cold PBS, and frozen at −80°C until purification.

#### Proximity biotinylation

The BioID protocol was adapted from Lambert et al. (2015) with slight modifications. Cell pellets from two 150-mm plates were pelleted, frozen, and thawed in 1.5 mL of ice-cold RIPA buffer containing 50 mM Tris-HCl (pH 7.5), 150 mM NaCl, 1% NP-40, 1 mM EDTA, 1 mM EGTA, 0.1% SDS, and 0.5% sodium deoxcycholate. PMSF (1 mM), DTT (1 mM), and Sigma-Aldrich protease inhibitor cocktail (P8340; 1:500) were added immediately before use. The lysates were sonicated, treated with benzonase, and centrifuged as described above. For each sample, 60 μL of streptavidin-sepharose bead slurry (GE Healthcare 17-5113-01) was prewashed three times with 1 mL of lysis buffer by pelleting the beads with gentle centrifugation and aspirating off the supernatant before adding the next wash. Biotinylated proteins were captured on prewashed streptavidin beads for 3 h at 4°C with rotation. The beads were gently pelleted and then washed twice with 1 mL of RIPA buffer and three times with 1 mL of 50 mM ammonium bicarbonate (pH 8.0). Following the final wash, the beads were pelleted and any excess liquid was aspirated off. Beads were resuspended in 100 μL of 50 mM ammonium bicarbonate, and 1 μg of trypsin solution was added. The samples were incubated overnight at 37°C with rotation and then an additional 1 μg of trypsin was added, followed by a further incubation for 2–4 h. The beads were pelleted and the supernatant was transferred to a fresh tube. The beads were rinsed twice with 100 μL of HPLC-grade water and the wash fraction was combined with the supernatant. The peptide solution was acidified with 50% formic acid to a final concentration of 2% and the samples were placed in a speedvac to dry. Tryptic peptides were resuspended in 25 μL of 5% formic acid and stored at −80°C until analyzed by mass spectrometry.

#### Experimental design for mass spectrometry experiments

For each analysis, two biological replicates of each bait were processed independently. These were analyzed alongside negative controls in each batch of samples processed. For BioID, cell lines expressing a BirA*-FLAG-GFP construct, a BirA*-NLS-FLAG construct, or no bait (i.e., empty cell line) were used. These control cell lines were grown in parallel to those expressing baits studied here and treated in the same manner (24-h tetracycline induction, etc.). To minimize carryover issues, extensive washes were performed between each sample (see details for each instrumentation type); and the order of sample acquisition on the mass spectrometer was also reversed for the second biological replicate to avoid systematic bias.

#### Preparation of HPLC columns for mass spectrometry

A spray tip was formed on a fused silica capillary column (0.75 μm ID, 350 μm OD) using a laser puller (program = 4; heat = 280, FIL = 0, VEL = 18, DEL = 200). Ten centimeters to 12 cm of C18 reversed-phase material (Reprosil-Pur 120 C18-AQ, 3 μm; Dr. Maisch HPLC GmbH) was packed in the column by pressure bomb (in MeOH). The column was then equilibrated in buffer A prior to sample loading.

#### Mass spectrometry acquisition using TripleTOF mass spectrometers

Five microliters of each sample was directly loaded at 400 nL/min onto the equilibrated HPLC column. The peptides were eluted from the column over a 90-min gradient generated by a NanoLC-Ultra 1D plus (Eksigent) nanopump and analyzed on a TripleTOFTM 5600 instrument (AB SCIEX). The gradient was delivered at 200 nL/min starting from 2% acetonitrile with 0.1% formic acid to 35% acetonitrile with 0.1% formic acid over 90 min followed by a 15-min cleanup at 80% acetonitrile with 0.1% formic acid, and a 15-min equilibration period back to 2% acetonitrile with 0.1% formic acid, for a total of 120 min. To minimize carryover between each sample, the analytical column was washed for 3 h by running an alternating sawtooth gradient from 35% acetonitrile with 0.1% formic acid to 80% acetonitrile with 0.1% formic acid, holding each gradient concentration for 5 min. Analytical column and instrument performance were verified after each sample by loading 30 fmol of BSA tryptic peptide standard (Michrom Bioresources, Inc.) with 60 fmol of α-Casein tryptic digest and running a short 30-min gradient. TOF MS calibration was performed on BSA reference ions before running the next sample in order to adjust for mass drift and verify peak intensity. The instrument method was set to a data-dependent acquisition (DDA) mode, which consisted of one 250-msec MS1 TOF survey scan from 400–1300 Da followed by twenty 100-msec MS2 candidate ion scans from 100 to 2000 Da in high-sensitivity mode. Only ions with a charge of 2+ to 4+ that exceeded a threshold of 200 cps were selected for MS2, and former precursors were excluded for 10 sec after one occurrence.

#### Data-dependent acquisition MS analysis

Mass spectrometry data were stored, searched, and analyzed using the ProHits laboratory information management system (LIMS) platform (Liu et al. 2016). Within ProHits, AB SCIEX WIFF files were first converted to an MGF format using WIFF2MGF converter and to an mzML format using ProteoWizard (v3.0.4468) and the AB SCIEX MS data converter (V1.3 beta). Thermo Fisher Scientific RAW mass spectrometry files were converted to mzML and mzXML using ProteoWizard (3.0.4468) (Kessner et al. 2008). The mzML and mzXML files were then searched using Mascot (v2.3.02) and Comet (v2012.02 rev.0). The spectra were searched with the RefSeq database (version 57, January 30, 2013) acquired from NCBI against a total of 72,482 human and adenovirus sequences supplemented with “common contaminants” from the Max Planck Institute (http://lotus1.gwdg.de/mpg/mmbc/maxquant_input.nsf/7994124a4298328fc125748d0048fee2/$FILE/contaminants.fasta) and the Global Proteome Machine (GPM; http://www.thegpm.org/crap/index.html). For the TripleTOF 5600 files, the database parameters were set to search for tryptic cleavages, allowing up to two missed cleavage sites per peptide with a mass tolerance of 40 ppm for precursors with charges of 2+ to 4+ and a tolerance of ±0.15 amu for fragment ions. Deamidated asparagine and glutamine and oxidized methionine were allowed as variable modifications. The results from each search engine were analyzed through TPP (the Trans-Proteomic Pipeline v4.6 OCCUPY rev 3) (Deutsch et al. 2010) via the iProphet pipeline (Shteynberg et al. 2011). SAINTexpress version 3.3 (Teo et al. 2014) was used as a statistical tool to calculate the probability value of each potential protein–protein interaction from background contaminants using default parameters. Unless otherwise specified, controls were compressed by half, to a minimum of eight controls, using a strategy first introduced by Mellacheruvu et al. (2013). Two unique peptide ions and a minimum iProphet probability of 0.95 were required for protein identification prior to running SAINTexpress.

#### MS data visualization and archiving

Functional enrichment analysis was performed using g:Profiler using the default parameters. Dot plots and heat maps were generated using ProHits-viz (https://prohits-viz.lunenfeld.ca) (Knight et al. 2017), while Venn diagrams were generated using Venny 2.1 (http://bioinfogp.cnb.csic.es/tools/venny/index.html) and refined using a local implementation of Chart Wizard (Google). All MS files used in this study were deposited at MassIVE (http://massive.ucsd.edu). They were assigned the identifier MSV000086613 and can be accessed at ftp://massive.ucsd.edu/MSV000086613. The password to access the files is “TBX2” until publication.

### Genomic data availability

ChIP-seq data sets using endogenous Tbx2-HA have been deposited in the NCBI Gene Expression Omnibus under superseries accession number GSE175705, with the ChIP-seq data available under GSE175703, and RNA-seq data available under GSE 174704. All bioinformatics analyses were carried out using publicly available packages as described above.

## Supplementary Material

Supplemental Material
