# Supplementary material for: TBX2 controls a proproliferative gene expression program in melanoma
Source: Genes Dev. 2021 Dec 1;35(23-24):1657–77. doi: 10.1101/gad.348746.121 (PMC8653791; doi:10.1101/gad.348746.121)
Supplement: Supplemental Material [file supp_35_23-24_1657__DC1.html]

TBX2 controls a proproliferative gene expression program in melanoma — Supplemental Material 

# TBX2 controls a proproliferative gene expression program in melanoma

## Supplemental Material

- Supplemental\_Table\_S1.xlsx
- Supplemental\_Table\_S2A.xlsx
- Supplemental\_Table\_S2B.xlsx
- Supplemental\_Table\_S3.xlsx
- Supplemental\_Table\_S4.xlsx
- Supplemental\_TABLE\_S5\_.xlsx
- Supplemental\_FIGURE\_S1\_R.tif
- Supplemental\_Figure\_S2S\_R.tif
- Supplemental\_FIGURE\_S3R.tif
- Supplemental\_Figure\_S4S\_R.tif
- Supplemental\_Figure\_S5R.tif
- Supplemental\_Figure\_S6R.tif
- Supplemental\_MatlMeth\_Legends.docx
