# Supplementary figures and images for: TBX2 controls a proproliferative gene expression program in melanoma
Source: Genes Dev. 2021 Dec 1;35(23-24):1657–77. doi: 10.1101/gad.348746.121 (PMC8653791; doi:10.1101/gad.348746.121)

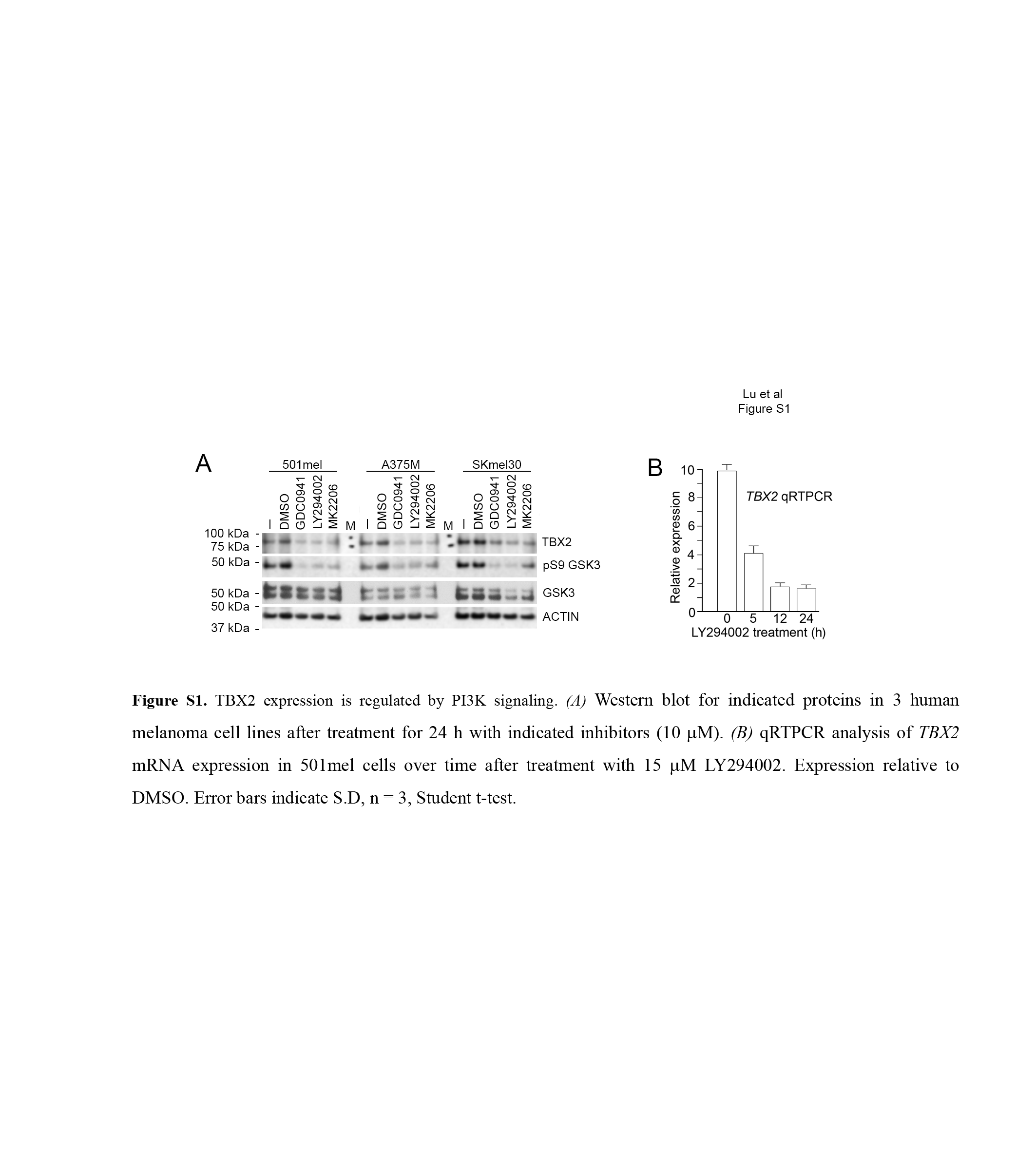

Supplement: Supplemental Material [file supp_gad.348746.121_Supplemental_FIGURE_S1_R.tif]

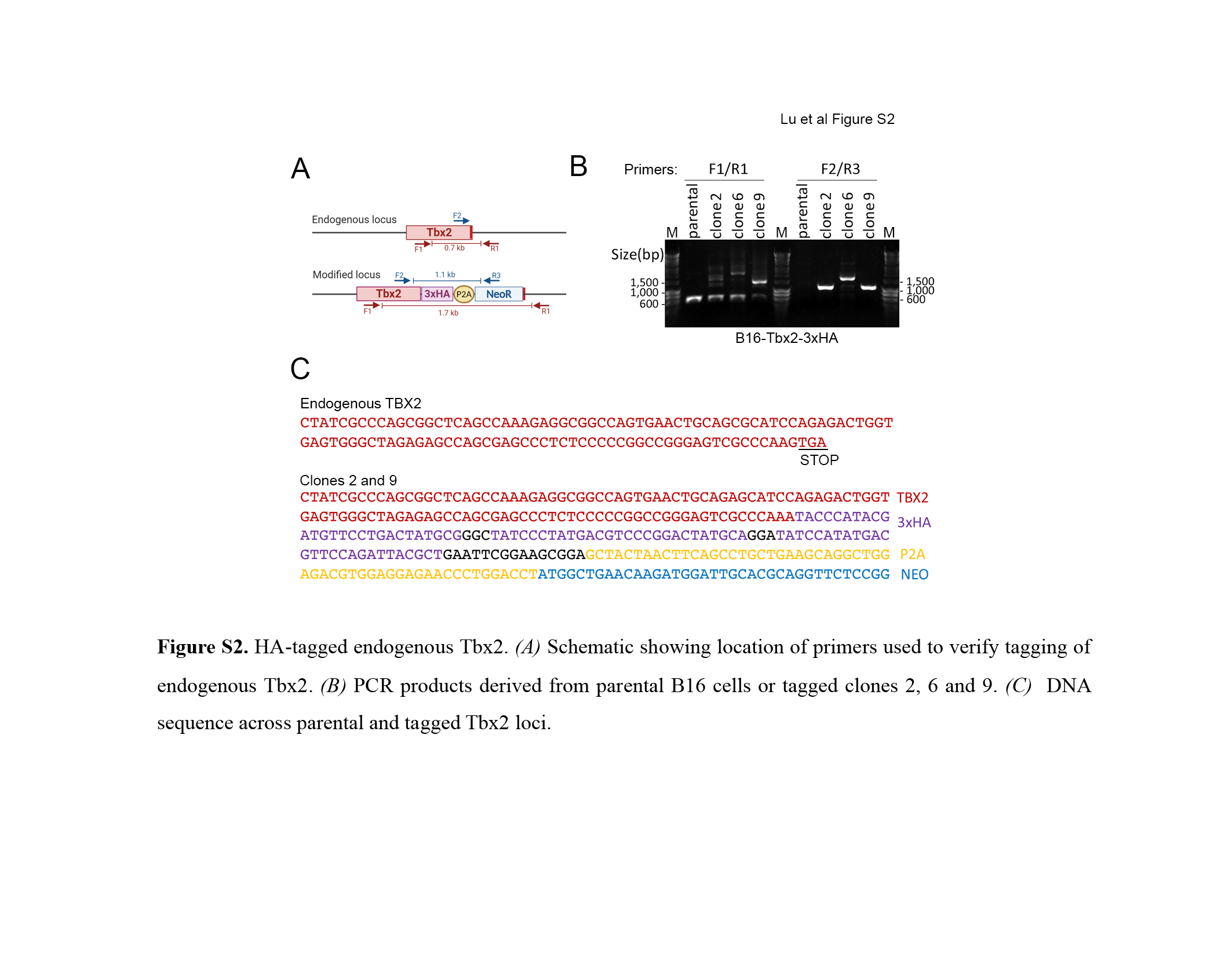

Supplement: Supplemental Material [file supp_gad.348746.121_Supplemental_Figure_S2S_R.tif]

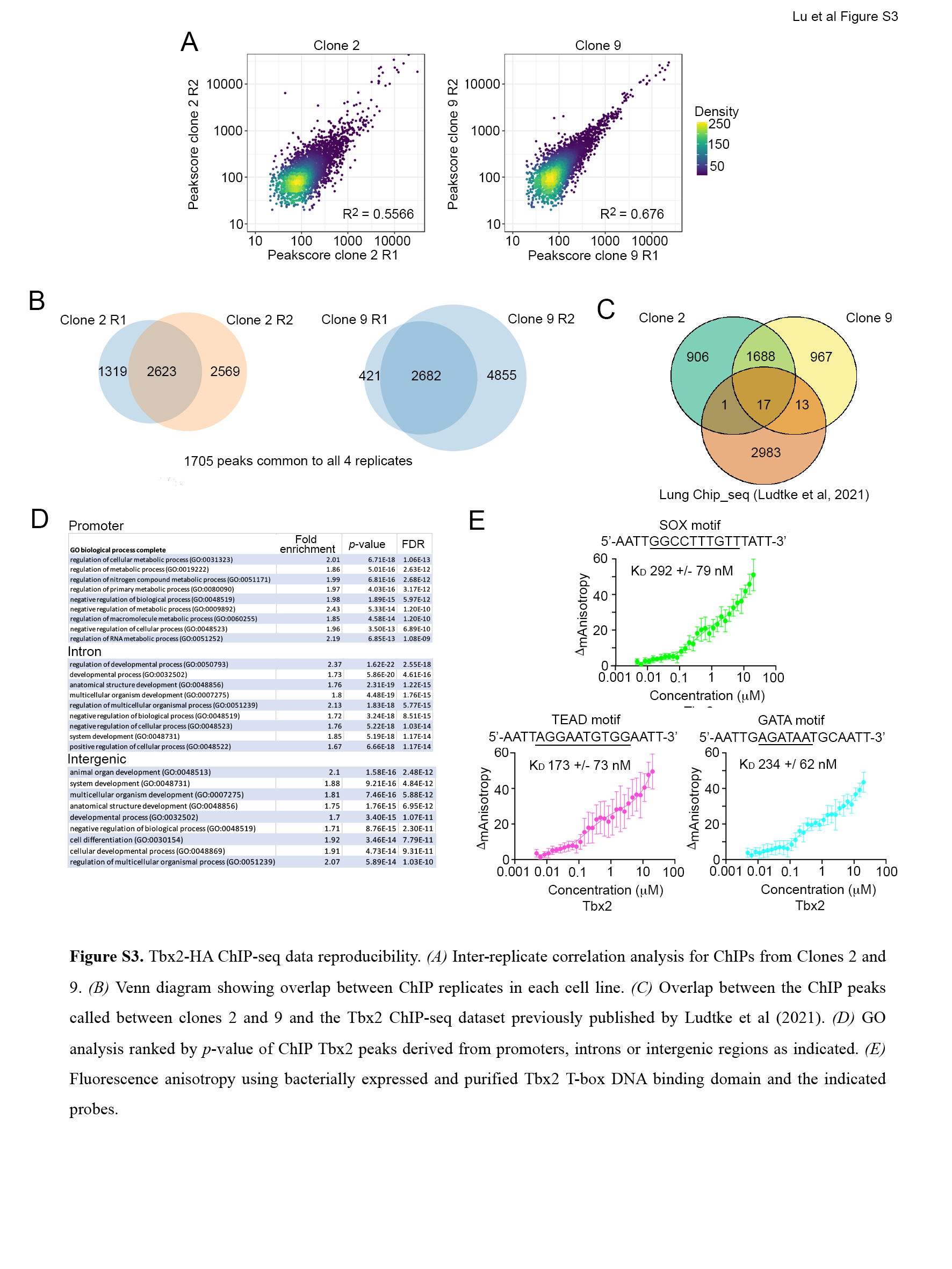

Supplement: Supplemental Material [file supp_gad.348746.121_Supplemental_FIGURE_S3R.tif]

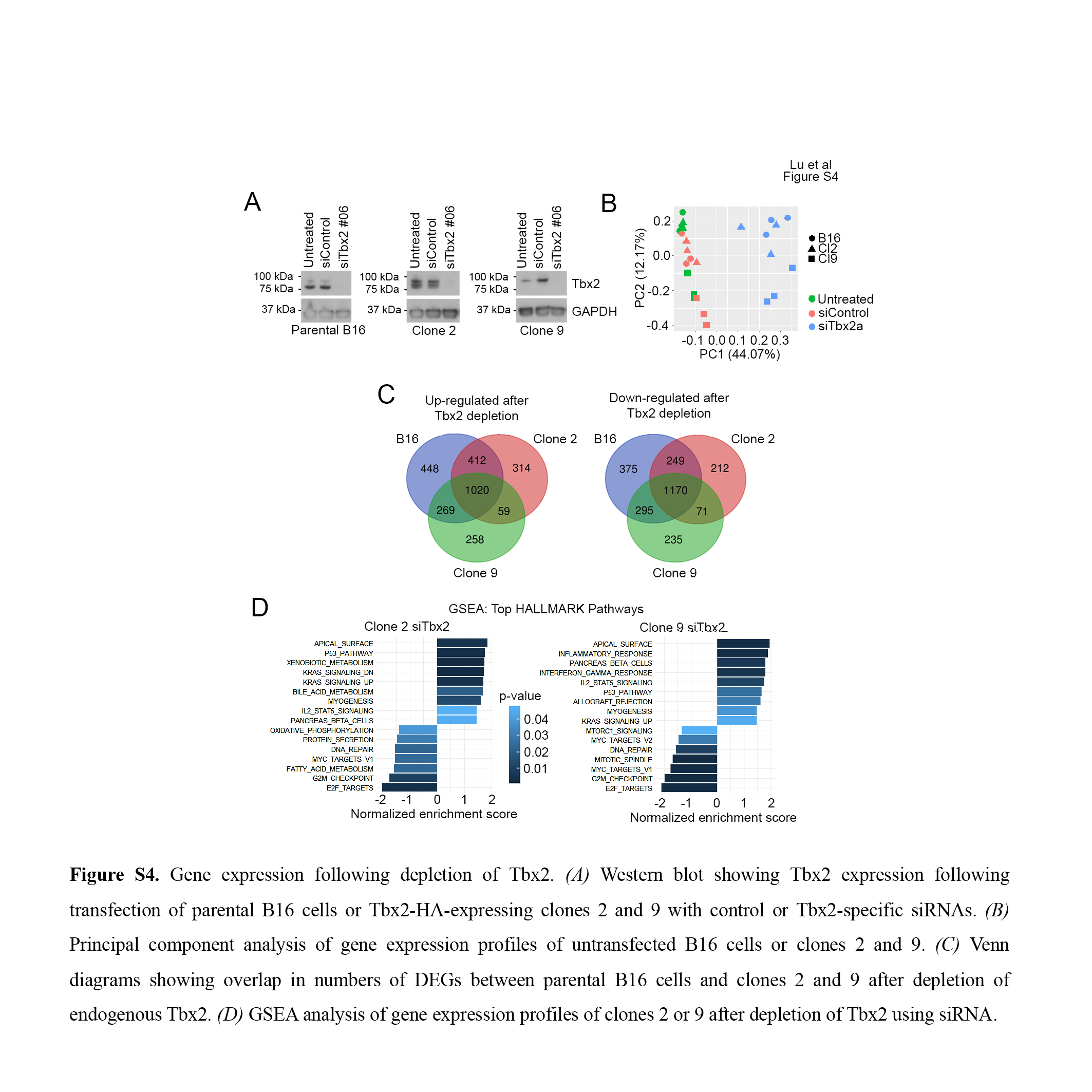

Supplement: Supplemental Material [file supp_gad.348746.121_Supplemental_Figure_S4S_R.tif]

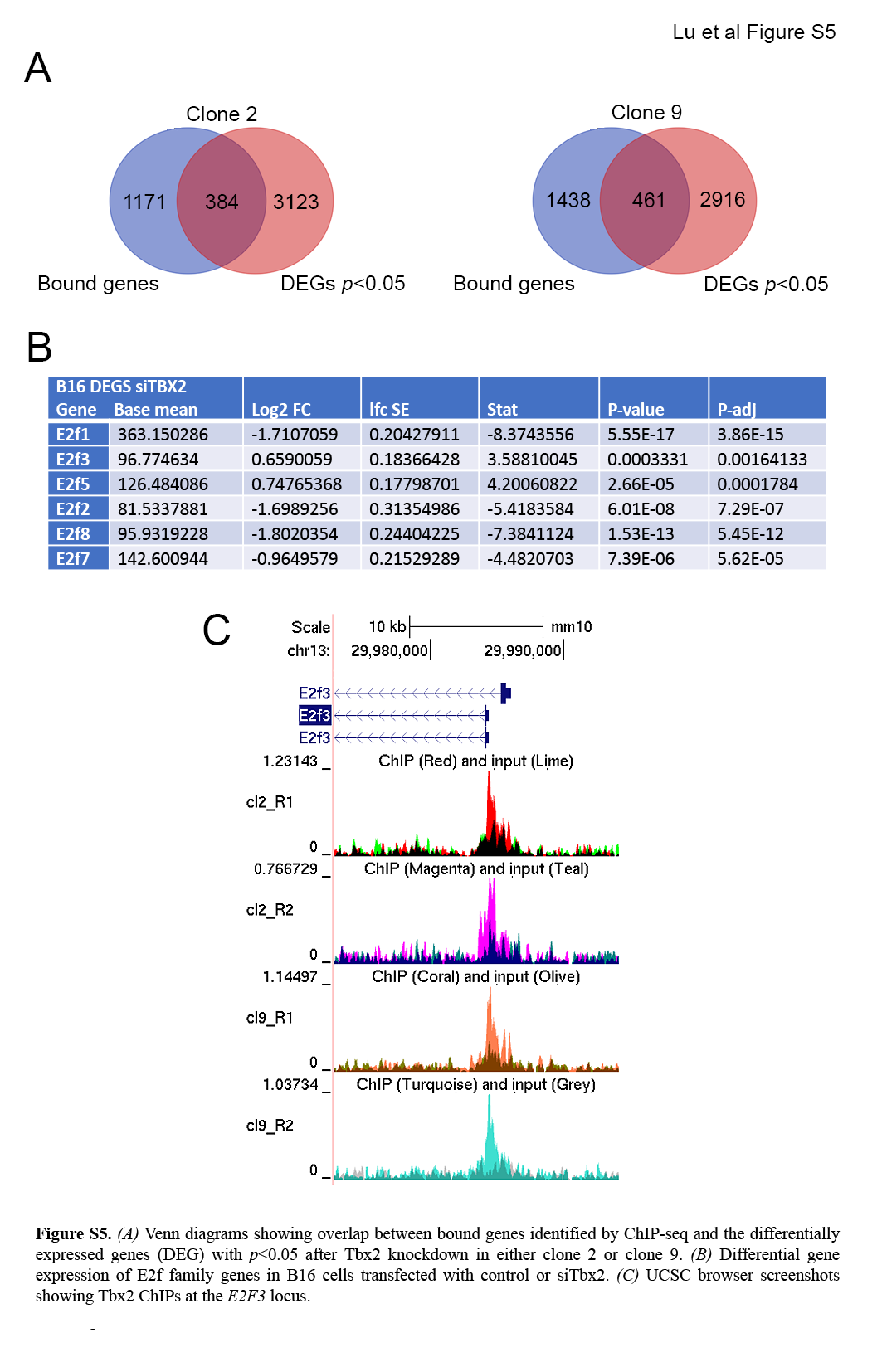

Supplement: Supplemental Material [file supp_gad.348746.121_Supplemental_Figure_S5R.tif]

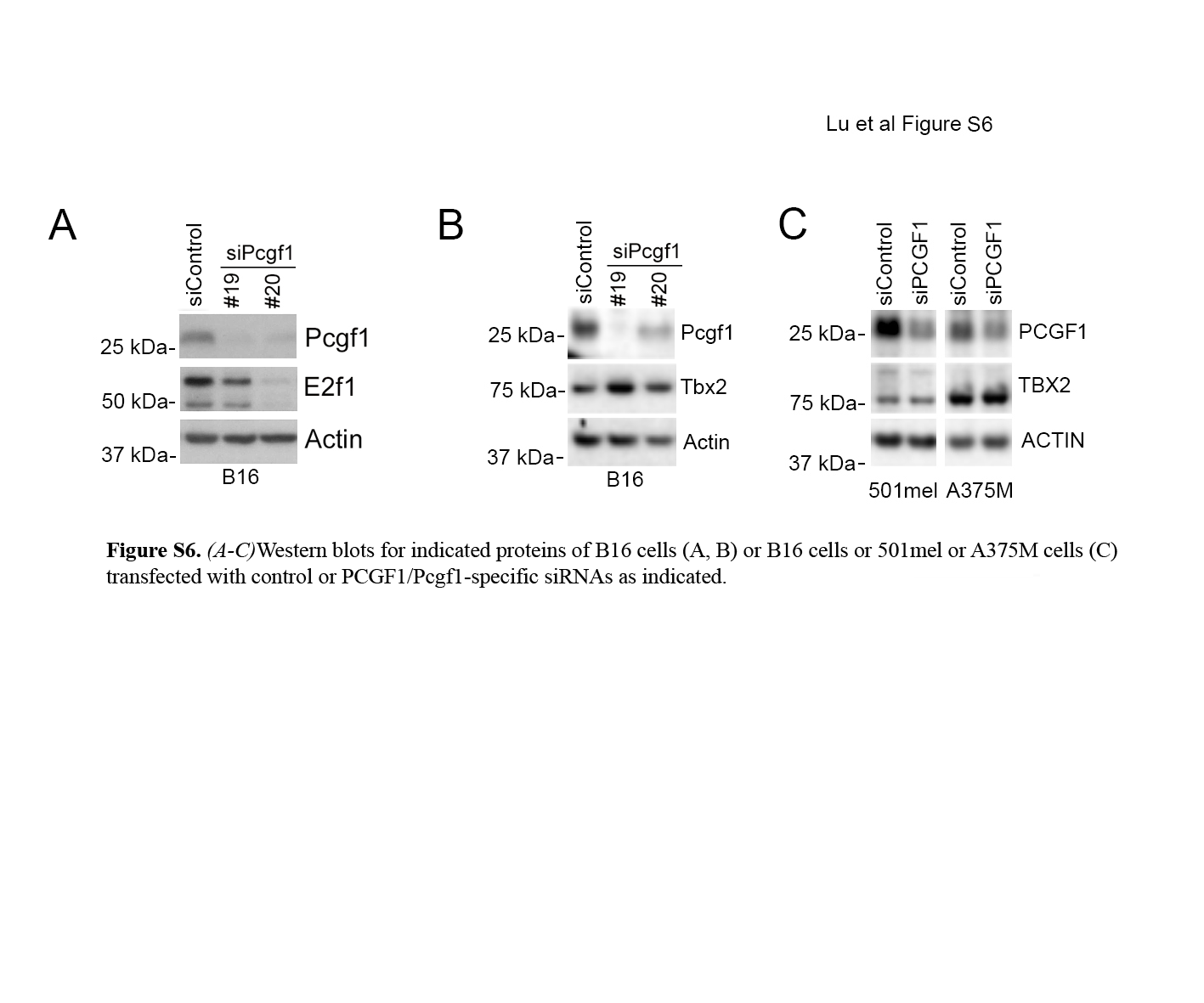

Supplement: Supplemental Material [file supp_gad.348746.121_Supplemental_Figure_S6R.tif]
