# Supplementary material for: TBX2 controls a proproliferative gene expression program in melanoma
Source: Genes Dev. 2021 Dec 1;35(23-24):1657–77. doi: 10.1101/gad.348746.121 (PMC8653791; doi:10.1101/gad.348746.121)
Supplement: Supplemental Material [file supp_gad.348746.121_Supplemental_MatlMeth_Legends.docx]

**SUPPLEMENTAL MATERIALS TABLE OF CONTENTS**

**Supplemental Materials and Methods**

**Supplemental Figure Legends**

**Supplemental Table legends**

**SUPPLEMENTAL MATERIALS AND METHODS**

*Table of antibodies, software, oligonucleotides, and siRNAs*

| **Antibodies** | | | |
| --- | --- | --- | --- |
| Actin | Sigma-Aldrich | | A4700 |
| ERK-2 | Santa Cruz | | Sc-1647 |
| E2F1 | Cell Signaling | | 3742 |
| CDK2 | Santa Cruz | | Sc-163 |
| GAPDH | Sigma-Aldrich | | G9545 |
| HA | Cell Signaling | | 3724 |
| HA (12C5) | Roche | | 11583816001 |
| Tbx2 | Home-made | |  |
| TBX2 | Sigma-Aldrich | | HPA008586 |
| PCGF1 | Santa Cruz | | sc-515371 |
| PCGF1 [EPR23757-38] | Abcam | | ab259943 |
|  |  | |  |
| **Genome Analysis Software and Algorithms** | | | |
| STAR | (Dobin et al. 2013) | |  |
| Deseq2 | (Love et al. 2014) | |  |
| ggplot2 | ISBN 978-3-319-24277-4 | | https://ggplot2.tidyverse.org. |
| Bowtie2 | (Langmead and Salzberg 2012) | |  |
| MACS2 | (Zhang et al. 2008) | |  |
| Homer | (Heinz et al. 2010) | |  |
| BedTools | (Quinlan and Hall 2010) | |  |
| seqMINER | (Ye et al. 2011) | |  |
| CistromeGO | (Li et al 2019) | | http://go.cistrome.org |
|  | | | |
| **Oligonucleotides** (sequence 5’→3’) | | | |
| AGGGCCTATTTCCCATGATTC | | IDT | SL056 |
| ATGTTCCTGACTATGCGGGC | | IDT | SL059 |
| AGCCCGGGTGGCCTGCGTGGCAGGGGAGCAGCTGGGCAGCCCCATGGGTGGAGGCGGTTC | | IDT | SL069 |
| AGCCAGCGAGCCCTCTCCCCAGGCCGGGAGTCGCCCAAATACCCATACGATGTTCCTGAC | | IDT | SL095 |
| TGCGCTTCAGTCCTTACCAG | | IDT | SL127 |
| CAGCATCCTCCACACCAGTT | | IDT | SL128 |
| GGTAGCCAACGCTATGTCCT | | IDT | SL132 |
|  | | | |
| Fluorescence anisotropy probes (sequence 5’→3’) | |  |  |
| AATTTCACACCTAGGTGTGAAATT **-**Fluorescein | | METABION |  |
| GAGAC*CACGTG*TTGAC **-**Fluorescein | | METABION |  |
|  | | | |
| **siRNAs** | | | |
| siCN:  MISSION® siRNA Universal Negative Control #1 | | Sigma | SIC001 |
| siTbx2 #05:  Sense (5’**→**3’) GCCAAAUACAUCCUGCUAAtt  Antisense (5’**→**3’) UUAGCAGGAUGUAUUUGGCtt | | Sigma | SASI_Mm01_00166394 |
| siTbx2 #06:  Sense (5’**→**3’) GGAUUUGAUGCAAAUUUCUtt  Antisense (5’**→**3’) AGAAAUUUGCAUCAAAUCCtt | | Sigma | SASI_Mm02_00315876 |
| siTbx2 #14:  Sense (5’**→**3’) CCAAAUACAUCCUGCUAAUtt  Antisense (5’**→**3’) AUUAGCAGGAUGUAUUUGGtt | | Sigma | SASI_MM02_00315877 |
| siTBX2 #15  Sense (5’**→**3’) CCAAUGAACUGCAGAGCAUtt  Antisense (5’**→**3’) AUGCUCUGCAGUUCAUUGGtt | | Sigma | SASI_HS01_00169003 |
| siTBX2 #16  Sense (5’**→**3’) CGCUAUAAGUUCCACAACUtt  Antisense (5’**→**3’) AGUUGUGGAACUUAUAGCGtt | | Sigma | SASI_HS01_00169004 |
| siPcgf1 #18  Sense (5’**→**3’) GCUUGCAAGACAGUGAAGAtt  Antisense (5’**→**3’) UCUUCACUGUCUUGCAAGCtt | | Sigma | SASI_MM02_00350685 |
| siPcgf1 #19  Sense (5’**→**3’) CCACUAUUAUCGAUAUGAUtt  Antisense (5’**→**3’) AUCAUAUCGAUAAUAGUGGtt | | Sigma | SASI_MM01_00075493 |
| siPcgf1 #20  Sense (5’**→**3’) GUCAUGCAGGACAUAGUGUtt  Antisense (5’**→**3’) ACACUAUGUCCUGCAUGACtt | | Sigma | SASI_MM01_00075494 |
| siPcgf1#21 | | Santa Cruz | sc-152107 |
| siBCoR | | Santa Cruz | sc-72636 |

**SUPPLEMENTAL FIGURE LEGENDS**

**Figure S1.** TBX2 expression is regulated by PI3K signaling. *(A)* Western blot for indicated proteins in 3 human melanoma cell lines after treatment for 24 h with indicated inhibitors (10 μM). *(B)* qRTPCR analysis of *TBX2* mRNA expression in 501mel cells over time after treatment with 15 μM LY294002. Expression relative to DMSO. Error bars indicate S.D, n = 3, Student t-test.

**Figure S2.** HA-tagged endogenous Tbx2. *(A)* Schematic showing location of primers used to verify tagging of endogenous Tbx2. *(B)* PCR products derived from parental B16 cells or tagged clones 2, 6 and 9. *(C)* DNA sequence across parental and tagged Tbx2 loci.

**Figure S3.** Tbx2-HA ChIP-seq data reproducibility. (A) Inter-replicate correlation analysis for ChIPs from Clones 2 and 9. (B) Venn diagram showing overlap between ChIP replicates in each cell line. (C) Overlap between the ChIP peaks called between clones 2 and 9 and the Tbx2 ChIP-seq dataset previously published by Ludtke et al (2021). (D) GO analysis ranked by p-value of ChIP Tbx2 peaks derived from promoters, introns or intergenic regions as indicated. (E) Fluorescence anisotropy using bacterially expressed and purified Tbx2 T-box DNA binding domain and the indicated probes.

**Figure S4.** Gene expression following depletion of Tbx2. *(A)* Western blot showing Tbx2 expression following transfection of parental B16 cells or Tbx2-HA-expressing clones 2 and 9 with control or Tbx2-specific siRNAs. *(B)* Principal component analysis of gene expression profiles of untransfected B16 cells or clones 2 and 9. *(C)* Venn diagrams showing overlap in numbers of DEGs between parental B16 cells and clones 2 and 9 after depletion of endogenous Tbx2. *(D)* GSEA analysis of gene expression profiles of clones 2 or 9 after depletion of Tbx2 using siRNA.

**Figure S5.** *(A)* Venn diagrams showing overlap between bound genes identified by ChIP-seq and the differentially expressed genes (DEG) with *p*<0.05 after Tbx2 knockdown in either clone 2 or clone 9. *(B)* Differential gene expression of E2f family genes in B16 cells transfected with control or siTbx2. *(C)* UCSC browser screenshots showing Tbx2 ChIPs at the *E2F3* locus.

**Figure S6.** *(A, B)*Western blots for indicated proteins of 501mel or A375M cells (A) or B16 cells (B) transfected with control or PCGF1/Pcgf1-specific siRNAs as indicated.

**SUPPLEMENTAL Tables**

**Table S1.** Annotated Tbx2 ChIP-seq peaks

**Table S2A.** Significant Differentially expressed genes obtained by RNA-seq of Tbx2-depeleted B16 cells

**Table S2B.** All Differentially expressed genes obtained by RNA-seq of Tbx2-depeleted B16 parental clone 2 and clone 9 cells

**Table S3.** Genes bound and regulated by Tbx2

Table S4. Cell cycle-related DEGs

**Table S5.** Mass spectrometry analysis of Tbx2-interacting factors identified by BioID.
